# Uncovering the hidden diversity of litter-decomposition mechanisms in mushroom-forming fungi

**DOI:** 10.1038/s41396-020-0667-6

**Published:** 2020-05-07

**Authors:** Dimitrios Floudas, Johan Bentzer, Dag Ahrén, Tomas Johansson, Per Persson, Anders Tunlid

**Affiliations:** 1 Department of Biology, Microbial Ecology Group, Lund University, Ecology Building, SE-223 62 Lund, Sweden; 2 Centre for Environmental and Climate Research (CEC), Lund University, Ecology Building, SE-223 62 Lund, Sweden

**Keywords:** Microbial ecology, Phylogenetics, Fungal ecology

## Abstract

Litter decomposing Agaricales play key role in terrestrial carbon cycling, but little is known about their decomposition mechanisms. We assembled datasets of 42 gene families involved in plant-cell-wall decomposition from seven newly sequenced litter decomposers and 35 other Agaricomycotina members, mostly white-rot and brown-rot species. Using sequence similarity and phylogenetics, we split the families into phylogroups and compared their gene composition across nutritional strategies. Subsequently, we used Raman spectroscopy to examine the ability of litter decomposers, white-rot fungi, and brown-rot fungi to decompose crystalline cellulose. Both litter decomposers and white-rot fungi share the enzymatic cellulose decomposition, whereas brown-rot fungi possess a distinct mechanism that disrupts cellulose crystallinity. However, litter decomposers and white-rot fungi differ with respect to hemicellulose and lignin degradation phylogroups, suggesting adaptation of the former group to the litter environment. Litter decomposers show high phylogroup diversity, which is indicative of high functional versatility within the group, whereas a set of white-rot species shows adaptation to bulk-wood decomposition. In both groups, we detected species that have unique characteristics associated with hitherto unknown adaptations to diverse wood and litter substrates. Our results suggest that the terms white-rot fungi and litter decomposers mask a much larger functional diversity.

## Introduction

Soils and plant biomass store an enormous amount of carbon in terrestrial ecosystems [1, 2]. These two carbon pools are linked with atmospheric CO_2_ via microbial litter- and wood decomposition [3, 4]. Large part of litter decomposition takes place in the upper layers of soil through the activity of litter decomposers (LD) found in mushroom-forming fungi. Many of those LDs are found in the order Agaricales (Basidiomycota) and are phylogenetically related to white-rot (WR) wood decayers [5].The phylogenetic proximity between the two groups in Agaricales is suggestive of a functional connection between litter and WR wood decomposition mechanisms. Similarly to WR fungi, LDs secrete diverse types of carbohydrate-active enzymes (CAZy) involved in carbohydrate degradation and oxidative enzymes involved in lignin degradation [6–11]. Some LDs cause preferential lignin degradation of leaf litter, a process termed as litter bleaching, which is considered to be the functional equivalent of WR [12–14]. By contrast, the equivalent of brown rot (BR) has not been reported for LD [15]. Despite the generic term used to describe them, LDs are found in diverse habitats (e.g., forested areas and grasslands) or substrates (e.g., fresh litter, humified organic matter, charred plant material, and dung) [16–18]. The decomposition mechanisms that underlie these adaptations remain unclear, but genome sequencing of a few LDs has revealed variations in the plant-cell-wall degradation (PCWD) machinery between species [19, 20].

Cellulose is a major carbohydrate of the PCW and is found in amorphous and crystalline form [21]. The latter form renders cellulose recalcitrant to microbial decomposition. Many fungi, including WR wood decayers, soft-rot wood decayers, and LDs, use extensive enzymatic systems to decompose cellulose [22]. These systems include enzymes that act on amorphous cellulose (e.g., endoglucanases) and enzymes that act on crystalline cellulose (e.g., cellobiohydrolases and lytic polysaccharide monooxygenases) [22, 23]. By contrast, enzymes acting on crystalline cellulose are largely absent from BR fungi [24]. These organisms use chelator-mediated Fenton-generated hydroxyl radicals that cause oxidative decomposition of carbohydrates and, to a smaller degree, lignin [25], which could explain the losses of lignocellulose genes seen in these fungi [24, 26, 27]. It has been shown that radicals attack amorphous cellulose early during BR decomposition [28], but there is less evidence of crystalline-cellulose modification by hydroxyl radicals [29–31].

Comparative genomics have revealed that transitions between nutritional strategies in fungi have left “signatures” in fungal genomes. Genomic signatures have been associated with the appearance of WR wood decomposition [24, 32], the parallel evolution of BR lineages [20, 24, 26, 27], and transitions from saprotrophic to ectomycorrhizal lifestyles [33]. More recently, such signatures have been identified in connection with parasitism and specialization to early wood colonization [34, 35]. LDs remain an evolutionary and functional black box, which limits our understanding of their role in the soil carbon cycle and their adaptation to diverse habitats.

We sequenced the genomes of seven LDs across three major clades in Agaricales and compared their core PCWD gene networks with those of 35 published genomes across mushroom-forming fungi. Our dataset includes two additional LDs; WR and BR wood decomposers; lignicolous species with uncertain types of wood decay (UWD) [20, 36]; and a mycoparasite. We hypothesized that LDs in Agaricales share to some extent the enzymatic machinery of WR fungi involved in PCWD. To compare the enzymatic machinery at the functional level, we focused on cellulose degradation and used Raman spectroscopy to examine structural changes in cellulose fibers after colonization by LDs, WR, and BR wood decayers. We hypothesized that LDs and WR species enzymatically depolymerize cellulose without affecting its crystallinity and that BR species disrupt the crystallinity of cellulose causing amorphogenesis.

## Materials and methods

Information about fungal strains, culture conditions, DNA / total RNA extraction, sequencing, genome assembly, and gene prediction can be found in the supplementary materials and methods.

### Data collection of plant-cell wall decomposition-related gene families

We collected data from seven newly sequenced genomes of LDs in Agaricales (Table S1) and 35 published genomes across 14 Agaricomycotina orders (Table S3) [20, 24, 27, 32, 33, 36–41]. We categorized the selected species into five nutritional strategies including LDs, WR and BR wood decayers, fungi with UWD types, and a mycoparasite [42–49]. We used IPR, Pfam, and SSF (superfamily) domain information [33, 50, 51] to collect data across 44 gene families related to PCWD [52] (see Supplementary methods, Table S4). We examined the collected protein models using alignment information excluding short or low quality models (the data have been deposited in Dryad, 10.5061/dryad.pk0p2ngk1). For the genomes of *Tetrapyrgos nigripes* and *Gymnopus confluens*, we additionally removed potential alleles (Fig. S1, 10.5061/dryad.pk0p2ngk1).

### Principal component analyses of gene families

**Fig. 1:**
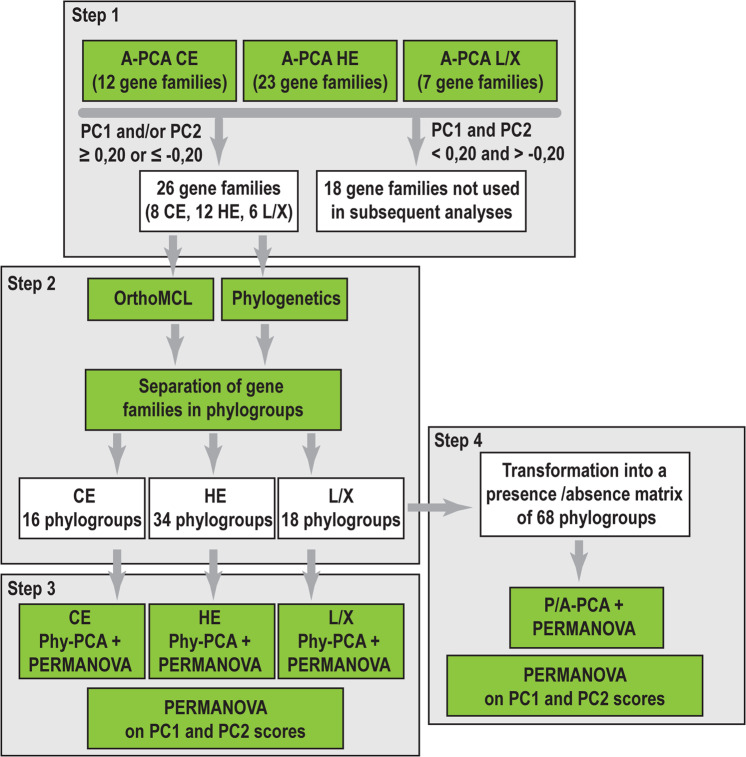
Methodology used for genomic data analyses. The 44 gene families associated with PCWD were split in three functional groups related to the degradation of cellulose (CE), hemicellulose and pectin (HE), and lignin/xenobiotics (L/X). For each functional group, a PCA analysis was performed (A-PCA) to select the families with the largest gene-copy variation across the 42 genomes (step 1, Table S6). The selected families were separated in phylogroups using OrthoMCL and phylogenetic analyses (step 2). For each functional category (CE, HE, and L/X), PERMANOVA analyses followed by a second series of PCAs were performed using the gene-copy numbers across phylogroups (Phylo-PCA, step 3, Table S6). The phylogroup data from all three functional groups was combined into one matrix and transformed into a presence/absence matrix (omitting copy number information per species within a phylogroup). PERMANOVA and PCA (P/A PCA) analyses were performed to examine the distribution of phylogroups across species (step 4, Table S6).

We split the PCWD dataset in three functional groups [52] containing gene families related to the enzymatic degradation of cellulose (CE, 12 families), hemicellulose and pectin (HE, 23 families), and lignin/xenobiotics (L/X, 7 families) (Tables S5). We analyzed the three datasets using principal component analyses (PCA) to identify the gene families that showed the largest gene-copy variation across species and across nutritional strategies (Fig. 1, step 1, A-PCA). For the gene families with PC1 and PC2 loadings ≤−0.20 or ≥ 0.2, we used phylogenetic and OrthoMCL [53] information to split them into phylogroups (Fig. 1, step 2). These analyses resulted in three new matrixes containing the gene-copy numbers for the 42 species across the generated phylogroups related to the degradation of cellulose, hemicellulose, and lignin/xenobiotics. We used the three matrixes to perform two different types of analyses. First, we performed one-way PERMANOVA to examine if they were significant differences in phylogroup gene composition between nutritional strategies and between the two most densely sampled orders of Agaricales and Polyporales, followed by pairwise comparisons (Bonferroni-corrected p-values) (Table S7). Second, we performed three new PCA analyses (step 3, Phylo-PCA) in order to identify those phylogroups that contributed the most to the separation of nutritional strategies. We then combined the three matrixes into one, which we converted into a presence/absence matrix in order to examine how the phylogroups are distributed between nutritional strategies and between the two most densely sampled orders of Agaricales and Polyporales. We preformed one-way PERMANOVA analysis on the presence/absence matrix, followed by pairwise comparisons using Bonferroni-corrected p-values and a PCA analysis (step 4, P/A-PCA). To further evaluate our findings, we performed one-way PERMANOVA analyses on the PC1 and PC2 scores of the species from all previous PCA analyses to identify significant differences on the distribution along PC1 and PC2 between the nutritional strategies and between the two most densely samples orders. Detailed information on phylogroup identification and the PCA settings can be found in the supplementary materials and methods.

### Modification of crystalline cellulose

We used Raman spectroscopy to examine the modification of crystalline cellulose after its colonization by the seven newly sequenced LDs, one WR (FD-574, *Phanerochaete* sp., dikaryon, Simlångsdalen, Halland, Sweden, available in our laboratory) and one BR (OMC-1627 *Gloeophyllum* sp., dikaryon, Puerto Rico, USA, deposited at University of Helsinki) species. In addition, we examined the modification of cellulose by NaOH, which transforms crystalline cellulose into its amorphous form [54], and by the commercial mixture of cellulolytic enzymes Cellic CTec2 (Novozyme). Detailed information on the culture conditions and the incubation of cellulose with NaOH and Cellic CTec2 is found in the supplementary material and methods.

### Raman data collection and analysis

We collected Raman cellulose spectra from cellulose samples colonized by fungi (three replicates) and samples treated with NaOH or Cellic CTec2 (seven replicates) (Table S8, Figs. S6 and S7) covering the spectra region from 1200 to 1550 cm^−1^. We analyzed the collected spectra using multivariate curve resolution–alternating least squares (MCR-ALS) [55]. We examined the Eigen values to select the number of components that were used to model the data and ran the MCR-ALS routine until convergence was reached. Additional information on data collection and analysis can be found in the supplementary materials and methods.

## Results

### PCWD gene families and identification of phylogroups

The genome size of the seven newly sequenced LDs (Table S1) was between 45 and 60 Mb (Table S2), except for the genome of *T. nigripes*, which was about 99 Mb. The final protein dataset for 44 PCWD gene families (Table S4) [52, 56] from the 42 genomes (Table S3) contained 7092 protein sequences (Table S5), which we sorted in three functional categories representing genes involved in cellulose, hemicellulose (including pectin), and lignin/xenobiotics degradation [52]. The comparison between the five nutritional strategies (WR, BR, UWD, LD, and MYC) with respect to the average number of copies for each category showed minor differences between LD and WR fungi (Fig. S2).

We performed the first set of three principal component analyses (termed here A-PCA, Fig. 1) using the gene-copy numbers across the CE, HE, and L/X datasets. This allowed us to identify the gene families that contribute more strongly to gene-copy number variation across the 42 genomes. In total, 26 of the 44 gene families (8, 12, and 6 gene families related to cellulose, hemicellulose, and lignin/xenobiotics, respectively) had PC1 and/or PC2 values ≥ 0.20 or ≤ −0.20. We chose those families for further analyses (Table S6).

**Fig. 2:**
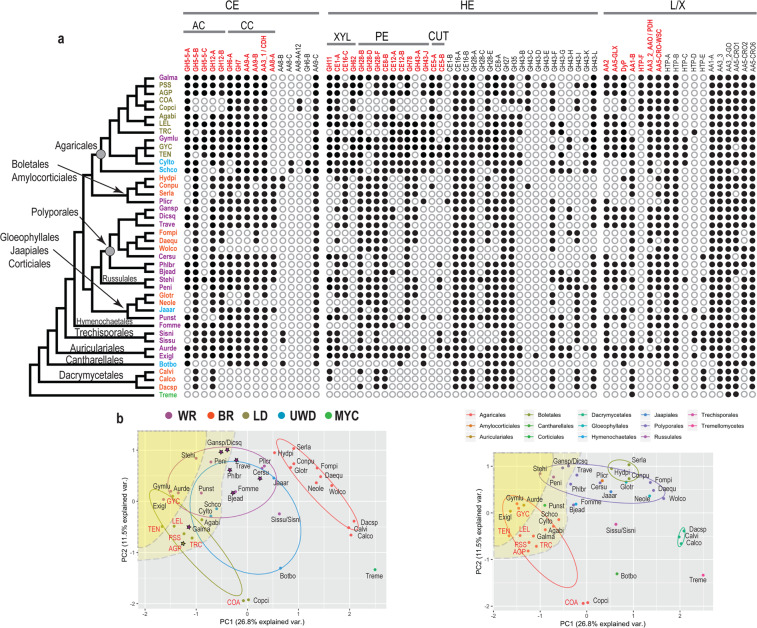
Phylogroup abundance patterns in mushroom-forming fungi. **a**. Presence (filled circles)/absence (unfilled circles) of 68 phylogroups across 42 species (species names can be found in Tables S1 and S3). The species tree is based on a phylogenomic tree published in Varga et al. [81]. Species acronyms colors are as in b. Phylogroup names in red indicate the phylogroups that have the strongest contribution according to the PCA analysis (≥0.15 or ≤0.15, Table S6). Phylogroups associated with the degradation of specific biopolymers are indicated by: CE (cellulose), AC (amorphous cellulose), CC (crystalline cellulose), HE (hemicellulose), XYL (xylan), CUT (cutin), and lignin/xenobiotics (L/X). **b** PCA plots from the P/A PCA analysis showing the grouping of the species based on nutritional strategy (left) and order classification (right). Species acronyms in red denote newly sequenced genomes. The yellow shaded areas include genomes that have genes in more than 75% (deep yellow) or more than 60% (light yellow) of the phylogroups. Species that code for high-redox potential Class II peroxidases (AA2) are shown with stars. Nutritional strategies: mycoparasite (MYC), brown-rot (BR), uncertain wood decay type (UWD), litter decomposer (LD), white-rot (WR). Species names can be found in Tables S1 and S3.

Many PCWD gene families are phylogenetically diverse, forming large subclades with different catalytic functions [57, 58]. To examine how gene-copy numbers within these subclades influenced the results from A-PCA, we used OrthoMCL and phylogenetic support (Fig. 1, Fig. S3, Table S10, 10.5061/dryad.pk0p2ngk1) to split the 26 gene families into 68 phylogroups, including 16 associated with cellulose, 34 with hemicellulose, and 18 with lignin/xenobiotics degradation (Table S6). Genes found within these phylogroups do not necessarily represent one-to-one orthologs [53], since internal gene duplications and losses may have taken place, but they represent homologs with high sequence similarity and presumably similar functions. The distribution of these phylogroups across the 42 analyzed species is shown in Fig. 2a.

### Enzymatic cellulose degradation is a conserved characteristic between LD and WR fungi

**Fig. 3:**
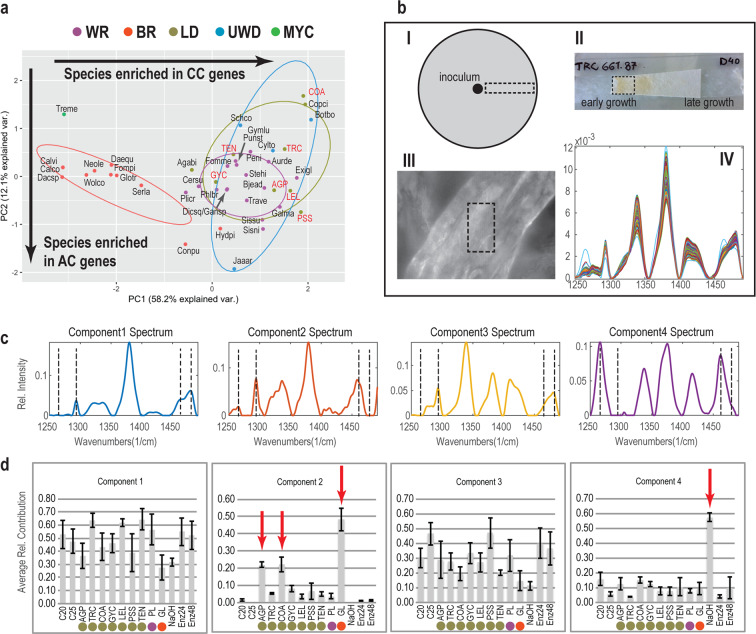
Distribution of cellulose decomposition genes and cellulose-degradation patterns across litter decomposing, white-rot, and brown-rot fungi. **a** PCA plot showing the distribution of cellulose genes from 16 phylogroups across 42 fungal species. CC: crystalline cellulose, AC: amorphous cellulose. The newly sequenced genomes are shown in red font. Species names can be found in Tables S1 and S3. Nutritional strategies: mycoparasite (MYC), brown-rot (BR), uncertain wood decay type (UWD), litter decomposer (LD), white-rot (WR). **b** Cellulose sample processing and Raman data collection. I-II. After incubation with fungi, NaOH or a commercial mix of cellulolytic enzymes (Cellic CTec2), a piece of the paper was radially excised and placed on a glass slide. III. A cellulose fiber under ×100 magnification. Raman data were collected from a rectangular area on the fiber. IV. Example of Raman data after area normalization, baseline correction, and smoothing using Savitzky–Golay (S–G) filtering. **c**. The four major Raman spectra components of cellulose resulting from MCR-ALS analyses. Black dashed lines indicate peaks related to crystalline and amorphous cellulose. **d** The average contribution of each component from panel c for each of the fungal samples, controls, enzymatic, or chemical treatments. Bars indicate standard error calculated from 198 or 126 spectra. Red arrows indicate species or treatments that transformed crystalline cellulose into amorphous. Colors indicate nutritional strategies as in **a**. C20, C25: control samples at 20 and 25 C, respectively; Enz24, Enz48: enzymatic treatment samples from the highest enzyme concentration at 24 h and 48 h, respectively.

We identified significant differences (PERMANOVA analyses, Table S7) between BR and all other nutritional strategies in terms of the constituent cellulose-degradation phylogroups. By contrast, we detected no significant differences between LDs, WR fungi, and fungi with UWD type, suggesting that the three nutritional strategies share the plesiomorphic enzymatic decomposition of cellulose. The Phylo-PCA for the cellulose-degradation phylogroups (Figs. 3a and S4a, Table S6) showed that phylogroups from families related to crystalline-cellulose degradation govern the distribution of species along PC1, whereas phylogroups from families related to amorphous-cellulose degradation govern the distribution of species along PC2, based on the PC loadings for the examined phylogroups (Figs. 3a and S4a, Table S6). The separation of BR fungi from other nutritional strategies was significant on PC1, which we attributed to the absence of most genes related to crystalline cellulose degradation (GH6, GH7, AA9, AA3_1-CDH) in BR fungi. We detected no significant differences between the four groups along PC2 (Table S7), which suggests that the endoglucanase content is independent of the nutritional strategy of the examined fungi. However, we observed a decreased number of endoglucanases for *Coprinellus angulatus* (COA, LD), *Coprinopsis cinerea* (Copci, LD), and *Botryobasidium botryosum* (Botbo, UWD) as compared with other species (Fig. S3b, c).

The large difference in copy numbers for genes governing crystalline-cellulose degradation between WR fungi /LDs and BR fungi pointed to fundamental differences in the mechanism involved in cellulose decomposition. To further examine these differences, we used Raman spectroscopy to analyze changes in the structure of crystalline cellulose caused by seven LDs, one WR fungus and one BR fungus (Fig. 3b). In addition, we examined separately the effects of a commercial preparation of cellulolytic enzymes and NaOH, which is known to dissolve crystalline cellulose [59]. MCR-ALS [55] analysis of all generated Raman spectra resulted in four major components that described more than 99% of the spectral diversity (Fig. 3c). The first and third components have characteristics of crystalline cellulose, represented by the peaks at 1481 cm^−1^ (methylene bending vibrations) and 1295 cm^−1^ (methylene twisting mode). The second and fourth components represent amorphous cellulose (or cellulose II), represented by the peaks at 1462 cm^−1^ (methylene bending vibrations) and around 1265 cm^−1^ (methylene twisting mode) [60–64]. Non-inoculated samples are mostly described by components 1 and 3, since the fibers consist mostly of crystalline cellulose I (Fig. 3d).

In agreement with the genomic data, the WR and five of the LD did not affect the crystallinity of cellulose (around 10% contribution of components 2 and 4 describe these treatments) (Fig. 3c, d). The enzymatic treatment at both enzyme concentrations resulted in the generation of glucose (Fig. S8), but similarly to the WR fungus and most LDs, we measured no changes in the crystallinity of the remaining fibers. These results suggest that WR fungi and LDs depolymerize cellulose chains without altering the crystallinity of cellulose in agreement with the enzymatic treatment and the genomic comparison between the two groups [65]. *C. angulatus* (COA) and *Agrocybe pediades* (AGP) were the only LDs that had an intermediate effect on cellulose crystallinity (Fig. 3c, d), and component 2 contributed about 20% to their spectra.

By contrast, the BR fungus *Gloeophyllum* sp. (GL) and the NaOH treatment caused large changes in cellulose crystallinity, with components 2 and 4 contributing about 50% to their spectra (Fig. 3c, d). While both *Gloeophyllum* and NaOH decreased cellulose crystallinity, the components describing these effects show differences. This is expected because fungal decomposition is a combination of factors acting on cellulose (chelator-mediated Fenton-generated radicals, metabolites, enzymes, and further modification/assimilation of generated products) [25], whereas NaOH only disrupts the crystalline structure of cellulose [59].

### Genomic signatures related to enzymatic hemicellulose and lignin degradation

**Fig. 4:**
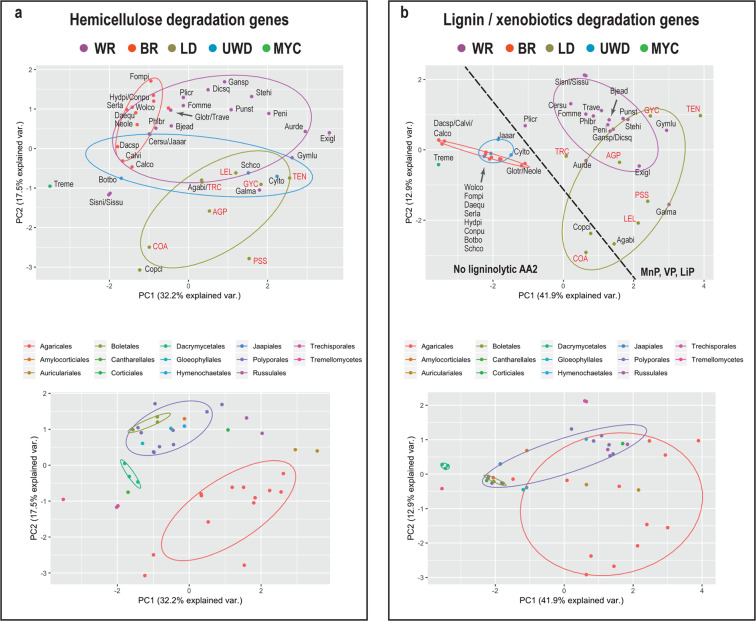
Differences of hemicellulose- and lignin degradation genes across nutritional strategies in mushroom-forming fungi. **a** PCA plots for 34 hemicellulose phylogroups showing grouping of species based on nutritional strategy (upper plot) and order classification (lower plot). Nutritional strategies: mycoparasite (MYC), brown-rot (BR), uncertain wood decay type (UWD), litter decomposer (LD), white-rot (WR). **b** PCA plots 18 lignin/xenobiotics phylogroups showing grouping of species based on nutritional strategy (upper plot) and order classification (lower plot). The newly sequenced genomes are shown in red font. The dashed line separates the species that lack ligninolytic Class II peroxidases (AA2) from ones that have at least one ligninolytic peroxidase (VP, MnP, LiP). Species names can be found in Tables S1 and S3.

We identified significant differences between BR fungi and all other groups, between WR fungi and LDs (Table S7), but also between Agaricales and Polyporales with respect to the gene content of hemicellulose- and pectin-degradation phylogroups. Phylo-PCA for the same dataset showed a small overlap between LDs and WR fungi (Fig. 4a, upper plot), which was mostly attributed to WR Agaricales species, and this became apparent when the species were grouped based on order classification (Fig. 4a, lower plot). BR fungi were significantly different from WR fungi and LDs along PC1, whereas LDs were significantly different from both WR and BR fungi along PC2 (Table S7). An examination of the loadings of the PCA variables suggested that the separation of LDs from WR and BR fungi was primarily attributed to xylan- (GH11, GH62, CE1-A), cutin- (CE5-A) and pectin-degradation (GH28-B, GH28-D) phylogroups. The separation of BR fungi from WR fungi and LDs could be primarily attributed to pectinesterase (CE8-A and CE12-A) and other pectin-degradation phylogroups (Fig. S4b, Table S6). Furthermore, we found that in contrast with other WR fungi or LDs, the species *C. angulatus* (COA)*, C. cinerea* (Copci)*, B. botryosum* (Botbo), and both *Sistotremastrum* species (Sissu, Sisni) coded for low number of genes across pectin-degradation phylogroups (e.g. GH78, CE8-B).

Similarly, we found that BR fungi and fungi with UWD types had significant differences for genes involved in the enzymatic degradation of lignin/xenobiotics from LDs and WR fungi, and we also found significant differences between LDs and WR fungi (Table S7). The Phylo-PCA for L/X degradation phylogroups showed that BR fungi and fungi with UWD types were significantly different along PC1 from WR fungi and LDs (Table S7). This difference was due to differences in class II peroxidases (AA2), laccases sensu stricto (AA1), dye-decolorizing peroxidases (DyP), glyoxal oxidases (AA5), and genes similar to aryl alcohol and pyranose dehydrogenases (AAO-PDH). The differences between WR fungi and LDs were significant along PC2 and were attributed primarily to differences in the gene content for HTP-F genes (heme-thiolate peroxidases) and class II peroxidases (AA2). The latter group is more widespread in WR fungi. A separate one-way PERMANOVA analysis showed that the distribution of ligninolytic AA2 genes between LDs and WR was significantly different (*p* = 0.004). In contrast to AA2 genes, HTP-F genes were found more frequently in LDs.

### Agaricales species carry a rich repertoire of PCWD enzymes partly shared with distant lineages in Agaricomycetes

To examine the distribution of phylogroups across species, we constructed a presence/absence matrix of the 68 identified phylogroups and analyzed it using PCA (P/A-PCA) (Figs. 1 and 2a) and one-way PERMANOVA. The pairwise comparisons revealed significant differences between LDs, WR and BR fungi (Table S7). PC1 and PC2 explained together only 38.3% of the data variation, however, the inclusion of PC3 explained only an additional 8% of the data variation and was not considered further. Thirty-two phylogroups (Table S6, PC1 and/or PC2 values ≥ 0.15 or ≤−0.15) had stronger contribution to the placement of nutritional strategies along PC1 and PC2 (Fig. 2b). We found that BR fungi were significantly different from all other groups along PC1 (Table S7), which we attributed to the absence of many phylogroups in BR fungi, e.g. GH7, GH5_5-A (Fig. 2b, left plot). BR fungi contained at least one gene in only 32.4–52.9% of the examined phylogroups. By contrast, WR fungi and LDs had at least one gene in 50% or more of the examined phylogroups. Despite this similarity, LDs were significantly different from WR fungi along PC2, which suggests that the two groups have retained genes across different phylogroups to some extent. Differences were detected across phylogroups involved in pectin (GH28-D, CE8-A, CE8-B), xylan (GH11, GH62), cutin (CE5-A), and lignin/xenobiotics (HTP-F) degradation genes. The overlap between LDs and WR fungi was small and attributed to WR fungi found in Agaricales or in distant orders (Fig. 2b right plot). Furthermore, most LDs had at least one gene in more than 60% of the examined phylogroups, which we did not observe in all WR fungi. WR fungi formed a gradient along PC1, pointing to the differential retention of phylogroups within the group (Table S6, Fig. S5). Some of the WR species at the lower spectrum of phylogroup abundance were rich in high-redox potential AA2 peroxidases (LiP, VP, Table S9, and Fig. S3r). Three groups of species had different phylogroup composition from all other LDs, WR, or BR fungi. These groups include *C. cinerea* (Copci) and *C. angulatus* (COA)*, B. botryosum* (Botbo), and both *Sistotremastrum* species (Sissu, Sisni). We attributed this to the absence of phylogroups related to hemicellulose degradation (all species) and to amorphous cellulose and lignin degradation ((*Coprinellus/Coprinopsis* (COA, Copci) and *B. botryosum* (Botbo)).

### CAZy families contain phylogroups with diverse distribution across species and across nutritional strategies

We found that phylogroups from different CAZy families that act on the same PCW polymer were either overrepresented, underrepresented (Phylo-PCA), or completely absent (P/A-PCA) within the same species or nutritional strategy, pointing to coordinated evolution for some phylogroups. For example, the xylan-degradation-related phylogroups CE1-A, GH62, and GH11 included many genes found in Agaricales and Auriculariales (Fig. S5, Table S6). Similarly, we found the Class II peroxidases (AA2), DyP, glyoxal oxidases (AA5-GLX), and putative aryl-alcohol oxidases/pyranose dehydrogenases (AAO-PDH), which are involved in enzymatic lignin degradation, to be more frequently absent from the same species (Table S6). Such absence was also observed with respect to GH6-A, GH7, AA9-A, AA9-B, and AA3_1-CDH (Table S6), which are involved in crystalline cellulose decomposition. These families have been shown previously to represent the genomic signatures that differentiate WR from BR fungi [24, 26, 27]. We found a co-occurrence of phylogroups with less understood roles, such as AA5-CRO-WSC, which had PC values similar to the ligninocellulolytic phylogroups referred above (Table S6). The precise role of these genes is unknown, although their upregulation during fungal wood decay has been reported [66, 67]. At the same time, we detected phylogroups within gene families with contrasting distribution between species or between nutritional strategies (Fig. S4, Table S6). For example, putative AAO genes (AA3-2) are overrepresented in Agaricales, whereas MOX genes (AA3-3) have a uniform distribution across most species. Similarly, among the GH43 phylogroups, GH43-L and GH43-G (Fig. S3o) are the ones that most clearly separate LDs and WR fungi from BR fungi. GH5-5-B genes are found in most species, but BR fungi selectively lack genes from phylogroup GH5-5-A (Fig. S3c). Taken together, our results suggest that phylogroups within CAZy families are subject to different selection processes. This could be the result of phylogroups in the same gene family having different enzymatic activities, or of phylogroups that have the same enzymatic activity but differential regulation [68].

## Discussion

The terms WR, BR, and LD have been traditionally used to separate saprotrophic mushroom-forming fungi based on the type of colonized substrate and the mechanism of decay. The mechanism of PCWD in LDs is frequently considered to be similar to that of WR fungi [12]; however, this has not been examined in detail in previous work. We sequenced and compared the genomes of seven LDs from Agaricales with published genomes from 14 orders across mushroom-forming fungi. Comparison of the genes associated with PCWD revealed that for all three functional categories examined (cellulose, hemicellulose, and lignin degradation), LDs were significantly different from BR fungi (Figs. 3a and 4). By contrast, we found that LDs and WR fungi showed both similarities and differences with respect to the composition of PCWD genes. LDs shared with WR fungi the plesiomorphic enzymatic network involved in crystalline- and amorphous-cellulose decomposition. In contrast with the similarities between LDs and WR fungi in regards to the enzymatic network involved in cellulose degradation, we found clear genomic signatures related to hemicellulose- and lignin degradation genes that separate LDs from most WR fungi. The key differences were related to the lower content of AA2 genes and the absence of high ligninolytic potential AA2 genes (VP, LiP) in most LDs as compared with WR fungi. However, we did not find large differences in the laccase and DyP content between the two groups. The large numbers of AA2 genes in WR fungi might not be necessary for efficient lignin decomposition in soil environments, and a moderate number of manganese peroxidases along with laccases might be sufficient agents of enzymatic lignin degradation [8, 69]. Notably, LDs are particularly rich in HTP-F genes. The expansion of HTP genes that has been previously documented in *A. bisporus* has been suggested as a possible adaptation to humic environments [41]. Here, we have found that these genes belong to phylogroup HTP-F, which is frequently overrepresented in many other LD, hinting at a possible yet unknown role in their lifestyle.

Additional large differences were detected for cutin- (CE5), xylan- (GH11, CE1-A, GH62), and pectin (GH28, GH43) decomposition genes. Endoxylanases are found in CAZy families GH10 and GH11, but the two families have different substrate specificities [70]. Whereas GH10 genes are widespread in saprotrophic Agaricomycetes, GH11 genes were found only in LDs and few wood decayers. In conjunction with the prominent presence of GH62 α-arabinofuranosidase and acetyl-xylan esterase (CE1) genes, our results suggest a high versatility of LDs towards xylan-enriched substrates. Primary cell walls of Poaceae monocotyledonous plants are enriched in arabinoxylans [21], and the more diverse gene content for enzymes that depolymerize this substrate could be associated with the successful expansion of Agaricales in grasslands. The prominent presence of cutinase genes in LDs points to further adaptations associated with the decomposition of leaves and other cutin-covered tissues (e.g. young stems and seeds). The greater variability in the recovered gene composition between LD and WR fungi could be driven by the structural diversity of PCW substrates. Whereas cellulose is a chemically conserved biopolymer across plant tissues, hemicellulose and lignin are chemically diverse between species or developmental stages within species [21].

**Fig. 5:**
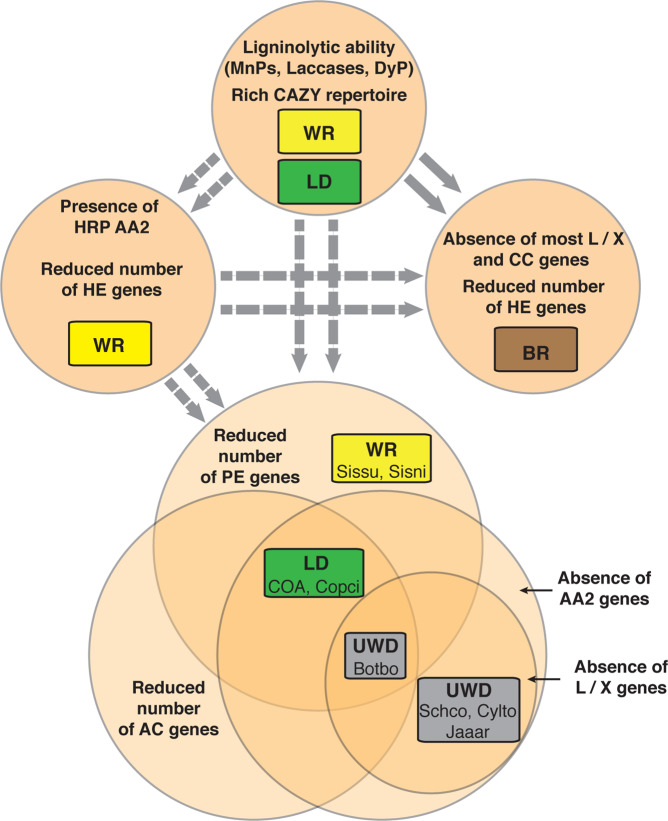
The discrepancy between recognized nutritional strategies and diversity of plant-cell-wall degradation (PCWD) genes across mushroom-forming fungi. The boxes indicate nutritional strategies (white-rot (WR), brown-rot (BR), litter decomposers (LD), uncertain wood-decay types (UWD)). The circles indicate distinct compositions of PCWD genes inferred from PC analyses. BR fungi fall into one PCWD gene composition, whereas WR fungi and LDs are found in more than one, which suggests that these strategies include species with diverse PCWD abilities. A subset of LDs and WR wood decayers are rich in PCWD genes, among them manganese peroxidases (MnPs), but lack high-redox potential (HRP) AA2 genes. A second set of WR species, which includes major wood decomposers (e.g. *Trametes versicolor*), has reduced content for genes related to hemicellulose degradation, but codes for diverse types of ligninolytic genes, including HRP AA2 genes (LiP, VP), which suggests adaptation to bulk-wood substrates. The Venn diagram represents three gene compositions with unique characteristics: reduced number or complete absence of genes acting on amorphous cellulose (AC), pectin (PE), and lignin. The latter group includes either a reduced number of ligninolytic genes (L/X) or an absence of ligninolytic AA2 genes. Distantly related species with different nutritional strategies (WR and LD, including the earlier recognized species of UWD) are found in the Venn diagram and share one or more of those gene compositions. This suggests that mushroom-forming fungi hide little-understood adaptations to PCW decomposition that have emerged multiple times. The dashed arrows suggest transitions from an ancestor rich in CAZy enzymes, whereas solid arrows indicate multiple transitions to BR from WR ancestors. Species names can be found in Tables S1 and S3.

Recent studies have questioned whether the placement of mushroom-forming fungi in guilds such as WR might mask the functional diversity of these fungi [20, 36]. Our work supports this contention: the gene networks involved in PCWD that we examined differ within guilds, which is suggestive of larger functional diversity than is currently thought to exist (Fig. 5). WR fungi formed a gradient in relation to the type and number of PCWD phylogroups, particularly in relation to hemicellulose degradation. This implies that over their long evolutionary history, the WR lineages have become functionally diverse and, therefore, WR species might not target the same PCW polymers or show similar carbohydrate-decomposition efficiency [71]. Similarly, even though sampling of LDs was restricted in Agaricales, LDs have variable gene composition related to lignin and hemicellulose degradation (Fig. 4). Furthermore, the results we present here take into consideration the enzymatic systems involved in carbohydrate and lignin degradation. However, non-enzymatic systems that could be active not only in BR fungi, but also across litter decomposing and WR fungi remain elusive at the genomic level. Therefore, we are still missing an important part of decomposition strategies that add to the diversity of PCWD strategies.

We also propose here that both LDs and WR fungi contain species with strongly deviating gene composition (Fig. 5). *C. angulatus* (COA), *C. cinerea* (Copci), *B. botryosum* (Botbo), and both *Sistotremastrum* species (Sissu, Sisni) lack most pectin decomposition genes. In addition, *B. botryosum* and *Coprinopsis*/*Coprinellus* lack AA2 genes and most amorphous-cellulose decomposition genes (*B. botryosum* lacks most other lignin-acting genes too). Our findings reveal that LD and WR include species that selectively lack certain parts of the PCWD machinery. These features could be related to shifts of hitherto unknown nutritional strategies [35, 36] or to a secondary-decomposer lifestyle, whereby substrates enriched in crystalline cellulose and depleted in hemicellulose and lignin are colonized. This suggestion agrees with previous work that has documented the frequent presence of *B. botryosum* and *S. niveocremeum* in well-rotten wood [72].

Phylogroups or gene families within a nutritional strategy can co-evolve (proliferation, retention, or gene loss) when the phylogroups or gene families participate in the decomposition of the same substrate (e.g. crystalline cellulose). At the same time, even within a single gene family, the evolution of phylogroups can be driven in opposite directions, such as in GH5_5, GH43, and GH28. Genes belonging to different phylogroups from the same gene family could be under different selective pressure, which suggests that they could have different functions and/or different regulation. Therefore, further separation of CAZy families in phylogroups offers a finer grained picture of gene-family evolution in connection with nutritional strategies in fungi.

Most LDs had high phylogroup abundance for PCWD gene families, which points to high versatility related to the decomposition of PCW carbohydrates. Only few WR fungi from Agaricales and distant orders such as Auriculariales, Corticiales, and Russulales showed similarly high phylogroup abundance. The presence of phylogroups across species from distant lineages suggests that the common ancestor of Agaricomycetes could have been rich in PCWD phylogroups, particularly with respect to genes related to hemicellulose decomposition (Fig. 5). The richest in phylogroup abundance species code exclusively for MnP genes, whereas WR species (and *A. pediades*) that are poorer in the examined phylogroups encode for high-redox potential AA2 genes (VP, LiP). This observation suggests that adaptation to strongly lignified substrates, such as bulk wood, could have resulted in a trade-off between the diversity of ligninolytic gene content and the diversity of CAZYmes, especially the ones related to hemicellulose degradation. While agreeing with previous work that has shown that VP and LiP genes evolved multiple times in Polyporales [73], our data suggests that this has also happened independently in Agaricales (Fig. S3r). Moreover, it explains reconstructions of ancestral AA2 genes, which previous studies have interpreted in terms of several amplification events of AA2 genes across WR lineages and a low number of MnP genes across the ancestral nodes of Agaricomycetes [24, 73, 74]. We found a similar composition in AA2 genes for species with high phylogroup abundance, which suggests that the common ancestor of Agaricomycetes could have been either a WR wood decayer or an LD with a rich set of CAZy enzymes that target diverse carbohydrates and a moderate number of AA2 genes (Fig. 5). Subsequent transitions to bulk-wood substrates could have given rise to more specialized saprotrophic organisms, such as WR species with strong ligninolytic potential, but also to the increasing number of little-understood saprotrophic strategies that we propose here (Fig. 5).

Previous work has shown that major shifts in nutritional strategies are associated with abrupt changes in the gene content of fungi. However, the extent to which we can learn about function by examining the gene content of fungi is a subject of debate [75]. By focusing on one aspect of PCWD, we show here that the genomic signatures we identified between LDs/WR fungi, and BR fungi correspond to functional differences in cellulose decomposition. Incubation of crystalline cellulose with commercial cellulolytic enzymes, WR fungi, or LDs had little effect on its crystallinity. This suggests that enzymatic degradation of crystalline cellulose is a peeling process, during which cellulose chains are successively degraded from the fiber’s surface without accumulation of amorphous material [65]. However, the system we have utilized here does not take into consideration the inhibition effect of lignin on the activity of enzymes. The intermediate effect of *C. angulatus* and *A. pediades* on the crystallinity of cellulose cannot be explained with the genomic data we present here, but as we mentioned earlier, the genomic data we present here cover mostly enzymatic mechanism of cellulose decomposition. Certain LDs and WR fungi might harbor additional enzymatic or non-enzymatic mechanisms involved in the modification of cellulose structure. The BR fungus *Gloeophyllum* generated cellulose with amorphous characteristics, which suggests that it disrupted cellulose crystallinity. This effect cannot be attributed to known enzymatic systems involved in cellulose decomposition, not only because BR fungi mostly lack those enzymes, but also because the enzymatic treatment did not result in the transformation of crystalline cellulose into its amorphous form. It has been suggested that a chelator-mediated Fenton reaction involving iron-reducing metabolites is responsible for the degradation of carbohydrate and, to some extent, lignin in BR fungi [25]. Most studies have documented the action of Fenton-based decomposition systems on amorphous cellulose [76], but only a few studies have reported the transformation of crystalline cellulose into its amorphous form [29–31]. We provide additional evidence for the transformation of crystalline cellulose into its amorphous form during BR decomposition at the fiber level. This suggests that rather than a peeling effect, BR fungi cause amorphogenesis of crystalline cellulose. Many enzymes that act on crystalline cellulose bind specifically on the surface of crystalline cellulose, either by catalytic center-cellulose interactions [77] or through the carbohydrate-binding module 1 (CBM1) [78, 79]. The transformation of crystalline cellulose into its amorphous form could hamper the binding ability of enzymes acting on crystalline cellulose [65, 78–80]. This, in turn, could render them redundant and could explain their preferential loss [24] along with the retention of endoglucanases across BR lineages.

## Supplementary information

Supplementary Material

## Data Availability

Assemblies are available from DDBJ/EMBL/GenBank under the following accessions: JAACJP000000000, JAACJO000000000, JAACJN000000000, JAACJM000000000, JAACJL000000000, JAACJK000000000, JAACJJ000000000. The original protein datasets, the aligned files and the phylogenetic trees have been deposited in Dryad (10.5061/dryad.pk0p2ngk1). The data that support the findings of this study are available from the corresponding author upon reasonable request.
