# Supplementary material for: Uncovering the hidden diversity of litter-decomposition mechanisms in mushroom-forming fungi
Source: ISME J. 2020 May 7;14(8):2046–59. doi: 10.1038/s41396-020-0667-6 (PMC7368018; doi:10.1038/s41396-020-0667-6)
Supplement: Supplementary file 1 — Supplementary Material [file 41396_2020_667_MOESM1_ESM.docx]

**Supplementary material**

**Table of contents**

**Supplementary Methods Page**

Newly sequenced fungal strains 3

Growth media used throughout the experiments 3

Cultivation of fungal strains for genomic DNA and total RNA isolation 4

Genomic DNA and total RNA isolation 5

PacBio genome sequencing and RNA sequencing 5

Genome assembly, mapping of RNA-Seq reads and genome annotation 6

Data collection and subclassification of protein models from the published

and newly sequenced genomes 7

Principal component analyses 8

Separation of proteins in phylogroups 9

Conditions for the modification of cellulose 12

Raman data analyses 13

Glucose measurements 14

**Supplementary Figures**

S1. Busco results for the seven newly generated genomes 15

S2. Average number of predicted proteins involved in the degradation

of hemicellulose, cellulose and lignin/xenobiotics for the five

nutritional strategies 16

S3. RAxML phylogenetic analyses for the 23 gene families used in

OrthoMCL, Phylo-PCA and P/A PCA analyses 17

S4. Variables coordinates from the phylogroups principal components

analyses for the cellulose, hemicellulose, and lignin/xenobiotics datasets 57

S5. P/A PCA variables coordinates 58

S6. Preprocessed Raman spectra of cellulose 59

S7. Cellulose spectra (1250-1550 cm^-1^) for samples colonized by the

*Phanerochaete* and *Gloeophyllum* strains 60

S8. Glucose generated from cellulose during Cellic CTec2 treatment 61

**Supplementary Tables**

S1. Newly sequenced fungal strains 62

S2. Genomic characteristics of the newly sequenced genomes 62

S3. Published genomes used in the study 63

S4. Pfam, IPR, SSF identifier used for data collection 64

S5. Gene copy numbers in 44 gene families across 42 genomes 65

S6. Putative enzymatic activity assigned to gene families and

PC values from A-PCA, Phylo-PCA, and P/A-PCA 65

S7. PERMANOVA analyses results 68

S8. Total number of replicates and number of Raman collected spectra

per fungal species, chemical and enzymatic treatments 69

S9. Subclassification of AA1, AA5, GH3, and AA3 across

the 42 genomes 69

S10. OrthMCL assignments for 23 gene families 69

**Supplementary files**

The following files are deposited in Dryad (doi.org/10.5061/dryad.pk0p2ngk1): a) the original protein files separated in those that were (group A) and those that were not (group B) used for OrthoMCL and phylogenetic analyses (.txt files), b) files containing those protein models removed after the initial alignments due to their low quality (those files have the format: FILENAME_excluded.txt), c) the final aligned files in PHYLIP format (.phy), d) the phylogenetic trees generated using RAxML.

**Supplementary Methods**

**Newly sequenced fungal strains**

We obtained seven strains of litter decomposers (Table S1) from the CBS-KNAW culture collection (<http://www.westerdijkinstitute.nl/collections>). The strains were routinely maintained on 2% malt extract (ME) agar growth medium. We tested the temperature preference of the strains by growing them on ME agar, oat extract (OE) agar and Fries medium (FM) agar at 20 and 25^o^C (Table S1). The ITS sequences of the seven strains have been deposited at GenBank under the accession numbers: MN121283-MN121289.

**Growth media used throughout the experiments**

**Malt extract growth medium** (**ME**). Malt extract 2% containing 10 ml/l vitamin stock solution (VM) and 1ml/l trace metal solution (TMS).

**Oat extract growth medium** (**OE**). Prepared by gently boiling 30 g oat flakes in 1 liter Milli-Q water for two hours, followed by filtration through a cloth, addition of 1 ml/l TMS and adjustment of the final volume to one liter.

**Trace metal solution** (**TMS**). 1 g ZnSO4.7H_2_O and 0.5 g CuSO_4_.5H_2_O in 100 ml distilled water. The addition of TMS is recommended to avoid irregular fungal growth.

**Vitamin stock solution** (**VM**). Per liter: 1g inositol; 0,01 g thiamine-HCl; 0,0025g biotin; 0,01g pyrodoxine; 0,01g riboflavine; 0,01g nicotinamide; 0,01g p-aminobenzoic acid; 0,01g Ca-panthotenate.

**Modified Fries growth medium (stock 1).** Per liter: 10g MgSO_4_.7H_2_O; 2g NaCl; 10g KCl; 1,5g H_3_BO_3_; 0,575g ZnSO_4_.7H_2_O; 0,125g CuSO_4_5H_2_O; 0,85g MnSO_4_. H_2_O; 0,02g (NH_4_)_6_Mo_7_O_24_.4H_2_O and 2,72 ml HCl 37%.

**Modified Fries growth medium (stock 2).** Per liter: 3g KH_2_PO_4_ and 2,72 ml HCl 37%.

**Modified Fries growth medium (stock 3).** Per liter: 2,6g CaCl_2_.2H_2_O and 2,72 ml HCl 37%.

**Modified Fries growth medium** (**FM & FM-N**). Per liter: 10ml of each stock solutions 1, 2, and 3; 10ml vitamin stock solution; 0,2g NH_4_Cl; 0,02g FeCl_3_.6H_2_O; 6g D-glucose, pH adjusted to 4,8. For FM-N, the nitrogen source was omitted.

**Highley growth medium** (**HG**). The growth medium contained per liter: 2g NH_4_NO_3_; 2g KH_2_PO_4_; 0,5g MgSO_4_.7H_2_O; 0,1g CaCl_2_.2H_2_O; 0,57mg HBO_3_; 0,036 mg MnCl_2_.4H_2_O; 0,31 mg ZnSO_4_.7H_2_O; 0,039 mg CuSO_4_.5H_2_O; 0,018 mg (NH_4_)_6_Mo_7_O_24_.4H_2_O; 0,015mg FeSO_4_.7H_2_O; 10 ml of vitamin stock (VM) solution.

**Soil organic matter** (**FH**). We collected soil from a Norway spruce stand in south Sweden, by removing the fresh litter layer followed by collection of the upper 10 cm of soil. We extracted the organic matter using hot water (1) (200 g soil / l water) and to the final extract we added 2,5 g/l glucose. Sterilization and particles removal was done by filtration (0,2 μm filter).

**Agar concentration.** For all culture conditions on solid media, we used 1,5 % agar.

**Cultivation of fungal strains for genomic DNA and total RNA isolation**

For genomic DNA isolation, each strain of the seven litter decomposers was inoculated in 250 ml Erlenmeyer flasks containing 50 ml ME medium. The cultures were incubated under static conditions at 20 or 25^o^C in darkness, except for the cultures of *L. leucothites*, which was incubated under shaking conditions (120 rpm). Once adequate mycelium had developed, we filtrated the mycelia, washed with autoclaved MilliQ water, froze them in liquid N_2_ and immediately stored them at -80 C.

For total RNA isolation, the seven strains were inoculated in six petri dishes (90 mm), respectively. Each petri dish contained a layer of acid-washed autoclaved glass beads and 10 ml of ME medium. For *P. cf. subviscida*, *L. leucothites*, and *T. nigripes* the glass beads were omitted because the fragility of the developing colonies made the collection of mycelia difficult. The cultures were incubated at 20 or 25^o^C. When about half of the petri dishes’ surface was covered with mycelia, the liquid medium was removed from three of the petri dishes, the mycelia were collected, washed with MilliQ water, immediately frozen in liquid N_2_, and stored at -80^o^C. For the remaining three petri dishes, the ME medium was removed, the petri dishes were washed with 10 ml of sterile MilliQ water and 10 ml of FM-N medium was added to induce nitrogen starvation. After 24 hours of incubation, the FM-N medium was removed, the petri dishes were washed with 10 ml of sterile MilliQ water and 10 ml of the FH extract was added. Incubation with FH lasted for 3, 6 or 9 days and the mycelia were harvested when the amount of glucose had reached below 40% of the initial concentration. For the fast growing strains, the mycelia were collected on day 3, while for the slow growing strains they were collected on day 9 (*T. constricta* & *G. confluens*). Following the same procedure described above, the mycelia were washed with MilliQ water, immediately frozen in liquid N_2_, and stored at -80^o^C.

**Genomic DNA and total RNA isolation**

Total genomic DNA was isolated using the Blood & Cell Culture DNA Maxi Kit (Qiagen, Cat No.: 13362) along with Proteinase K (Qiagen, Cat No.: 19133) and RNase A (Cat No.: 19101) treatments. The mycelia were thoroughly pulverized using a mortar and a pestle with addition of liquid N_2_ and DNA isolation was performed following the tissue samples protocol provided by the manufacturer. DNA was eluted in sterile water provided by the manufacturer and an additional cleaning step was performed using the Power Clean DNA Clean Up kit (MoBio, Cat No.: 12877-50), following the manufacturer’s instructions. We examined DNA quality and quantity using agarose-gel electrophoresis and NanoDrop 2000 and stored the samples at -80^o^C.

Total RNA was isolated using the RNeasy Plant Mini Kit (Qiagen, Cat No.: 74904), following the ‘Total RNA from plant cells and tissues and filamentous fungi’ protocol, with the addition of an on-column DNase digestion step (RNase-Free DNase Set, Qiagen, Cat. No.: 79254). RNA was eluted in RNase-free water provided by the manufacturer and stored at -80^o^C. The quality and quantity of the total RNA samples were assessed using Bioanalyzer (Agilent) and we shipped for most samples a final concentration of 90 ng/μl total RNA (volume: 25 μl) for sequencing.

**PacBio genome sequencing and RNA sequencing**

The PacBio libraries were produced using the SMRTbell™ Template Prep Kit 1.0 according to manufacturer’s instructions. In brief, 10 µg of genomic DNA per library were sheared into 20 kb fragments using the Megaruptor system (Diagenode), followed by an exo VII treatment, DNA damage repair and end-repair before ligation of hair-pin adaptors to generate a SMRTbell™ library for circular consensus sequencing. The library was then subjected to exo treatment and PB AMPure bead wash procedures for clean-up before it was size selected with the BluePippin system with a cut-off value of 9000 bp. One SMRTcell™ with library was sequenced on the PacBio RSII instrument using the P6-C4 sequencing chemistry and 600 minute movie time.

For RNA sequencing, Illumina TruSeq Stranded mRNA libraries were constructed with Poly-A selection. Clustering was done by 'cBot' and samples were sequenced on HiSeqX (HiSeq Control Software 2.2.58/RTA 1.18.64) with a 2x126 setup using 'HiSeq SBS Kit v4' chemistry. The Bcl to FastQ conversion was performed using None from the CASAVA software suite. The quality scale used is Sanger / phred33 / Illumina 1.8+. Standardized bioinformatics quality control checks were performed, which included checking the yield, sequence read quality and cross-sample contamination.

**Genome assembly, mapping of RNA-Seq reads and genome annotation**

Genomes of monokaryotic species were assembled by SciLifeLab using HGAP3 (2). We assembled the genomes of dikaryotic species using the PacBio FALCON assembler (3), followed by a polishing step of the assemblies using the RS_Resequencing protocol in PacBio SMRT portal (version 2.3.0). We examined the quality and the completeness of the polished genome assemblies using QUAST (4.4) (4) and BUSCO (2.0) (5) (Fig. S1). For the latter, we used the fungi basidiomycota_odb9 dataset.

The FastQC tool (www.bioinformatics.babraham.ac.uk/projects/fastqc/) was used to assess the quality of mRNA reads. The *hisat2-build* tool was used to build a HISAT2 index of the assembly FASTA file and the stranded pair-end reads were aligned to the primary and associated contigs using HISAT2 (2.0.6) (6) with the following parameters: --max-intronlen 5000--rna-strandness RF --dta. The resulting SAM files were sorted and converted to BAM files and mapping statistics were retrieved from the BAM files using SAMtools (1.3.1) (7). The transcripts were assembled using StringTie (v1.3.0b) (8).

Ribosomal RNA gene models were predicted using RNAmmer (1.2) (9). We masked repeats using RepeatMasker (10) and RepeatRunner (11) in MAKER2 (2.31.8) (12) using fungi as model organism in RepBase. Genes were also predicted with MAKER2 (2.31.8) using the StringTie assembled transcripts and the following gene prediction tools: the *-fungus* setting of GeneMark-ES (4.32) (13), AUGUSTUS (3.2.2) (14), and SNAP (15). SNAP was trained using MAKER2 and AUGUSTUS using BRAKER1 (1.9) (16). For the latter, the BAM files were merged from HISAT2 using SAMTools (1.3.1) and the merged BAM file was used as input to GeneMark-ET (13) to create a training set for AUGUSTUS. In BRAKER1 the *-fungus* setting in GeneMark-ET was used.

The predicted proteins were annotated using PfamScan (1.5) (17) against the Pfam (30.0) (18) database and InterProScan (5.27-66) (19) with the following databases: TIGRFAM, SFLD, SUPERFAMILY, ProSiteProfiles, SMART, CDD, PRINTS, ProSitePatterns, Pfam, ProDom and SignalP_EUK. The final predicted proteins had support from either RNA-Seq data or Pfam annotation or InterProScan annotation or a combination of those.

**Data collection and subclassification of protein models from the published and newly sequenced genomes**

For several gene families the use of Pfam or IPR domain identifiers was sufficient to collect the data (Table S4). In addition, the collected data was compared to the CAZy annotation of the JGI database and annotations from an earlier published study (20). For the gene families reported below additional strategies were used in order to verify the number of gene copies belonging to a family, and subclassify them.

**GH3, AA1, AA2, AA3, AA5**. After retrieving the data using the corresponding identifiers (Table S4), alignment/phylogenetic information and published annotated sequenced were used to subclassify these datasets. GH3 sequences were subclassified into putative β-glucosidases, β-xylosidases, and β-N-acetylhexosaminidases (21), AA1 sequences into laccases sensu stricto, laccase-like proteins, Fet3 reductases, melanin synthesis-related proteins, and L-ascorbate oxidases (22) (Fig. S3q), and AA2 sequences into cytochrome c peroxidases, L-ascorbate peroxidases and Class II peroxidases (Fig. S3r). Class II peroxidases were further subclassified into manganese (MnP), lignin (LiP), versatile (VP), atypical MnP, atypical VP, and generic (GP) peroxidases by aligning the dataset and identifying the LRET tryptophan, and the three manganese binding amino acid residues (23). For 21 AA2 sequences certain subclassification could not be provided because of gaps in the sequence. The GMC proteins (AA3) were separated into potential glucose oxidase, aryl alcohol oxidase/ pyranose dehydrogenases, methanol oxidases, cellobiose dehydrogenases or proteins with uncertain classification (24) (Fig. S3v). The GMC proteins represent the most challenging gene family to further subdivide into functional categories and the grouping of genes was done to minimize leaving out proteins that could be related to a certain group. However, the classification here should be seen only as putative particularly for the category of glucose oxidases, while methanol oxidases and cellobiose dehydrogenases are easy to classify based on phylogenetic information. The AA5 were subclassified into CRO1, CRO2, CRO6, GLX and CRO-WSC (CRO3-5) (25) (Fig. S3u). The gene copies distribution across all species after subclassification of AA1, AA2, AA5, and GH3 can be seen in Table S9.

**GH2, GH27, GH5-5/GH5-7, GH74, GH78, GH88, GH95, CE12/CE16**. For these gene families in addition to identifier information annotated data from JGI and alignment information were used in order to retrieve the proteins that belong in the families, because the identifiers used represented a larger pool of proteins with distant homology.

For the following families blastp searches were also used to retrieve data:

**AA8, AA8/AA12**. For the published genomes the protein model Trave1_45408 was used in blastp searches to recover the AA8 and AA8/AA12 proteins. For the newly sequenced genomes the term SSF49344 was used, which recovers AA3_1, AA8, AA12, but also other distant proteins that belong to this superfamily. AA8 and AA12 proteins were separated from the larger dataset using alignment information.

**CE1**. The term IPR010126 in addition to blast searches using the protein Gymlu1_ 198053 were used for all the published genomes. The use of the Gymlu sequence was necessary because the IPR domain is not present in one of the two major clades of CE1 proteins. The SSF53474 identifier was used for the newly sequenced genomes followed by separation of CE1 proteins using alignment information.

**GH115**. For the new genomes the Pfam identifier PF15979 was used to recover the predicted GH115 proteins and for the published genomes GH115 annotated proteins (20) were used for blastp searches.

**Principal component analyses**

The dataset was split in three functional groups containing genes related to the degradation of cellulose (CE, 12 families), hemicellulose and pectin (HE, 23 families) and lignin/xenobiotics (L/X, 7 families) (Table S5). For each functional group, the gene copy number information from each gene family across the 42 genomes was used to perform a principal component analysis (PCA) (A-PCA, Fig. 1) and identify the families with PC1 and PC2 loadings ≤ -0,20 or ≥ 0,2. (Table S6). For each of these families, OrthoMCL (26) was used to identify orthologous groups (Table S10) and a phylogenetic tree using RAxML was constructed to identify strongly supported phylogenetic clades (see below, Fig. S3 a-v). For each functional category (CE, HE and L/X), the distribution of genes in phylogroups was used to perform PCA (termed here Phylo-PCA, Fig. 1, Table S6). Finally, the phylogroup data from all three functional groups were combined into one matrix and transformed into a presence-absence matrix (omitting copy number information per species within a phylogroup). The differences of the species based on the presence-absence patterns of phylogroups was examined using PCA (P/A PCA, Fig. 1, Table S6).

All PCAs were done in RStudio (1.1.456). First, all values of copy numbers were increased by one in order to remove zero values from the matrixes. The values were log_2_ transformed to take into account large variation in copy numbers within gene families. The selection of components was done by examining the Eigen values from each PCA. The PCA plots were generated with ggbiplot ([https://github.com/vqv/ggbiplo](https://github.com/vqv/ggbiplo)). Normal contour lines (68% probability) were drawn based on nutritional strategies or order classification.

**Separation of proteins in phylogroups**

Separation of gene families in phylogroups was done using a combination of phylogenetic support and OrthoMCL protein clustering. The default settings were used in OrthoMCL (26) to group the protein sequences and phylogenetic analyses were performed using RAxML in CIPRES (27,28). Each protein dataset was aligned using MAFFT (FFT-NS-i) (29), the resulting alignments were examined using AliView (30) and poorly aligned regions such as the signal peptide, linkers, and CBM1 were removed. For the phylogenetic analyses PROTCAT and WAG as the amino acid substitution matrix were used and 1000 rapid bootstrap runs were performed followed by a maximum likelihood (ML) search. The resulting trees can be seen in Fig. S3, while the files containing the proteins from each family, the final alignments used for phylogenetics, and the tree in Newick format can be found in File S2.

The OrthoMCL results were mapped on the phylogenetic trees and we examined conflict between phylogenetic clades (bootstrap support ≥ 80) and protein clustering. For the gene families: AA8, CDH, CE5, CE8, CE12, GH5-5, GH6, and GH12 OrthoMCL protein grouping was in complete agreement with well-supported phylogenetic clades. For the rest of the datasets, when there was conflict between OrthoMCL and phylogenetic clades, phylogenetic support was prioritized as the criterion for generation of phylogroups. For those proteins that OrthoMCL could not place them in any orthogroup (no group) (26), it was examined whether the proteins had the same best blast hit in the OrthoMCL results and simultaneously belonged to the same phylogenetic clade. In this case, these proteins were recognized as a phylogroup. When neither OrthoMCL nor phylogenetic support allowed the separation of proteins in phylogroups then the family was treated as a phylogroup. In detail:

**AA1.** The majority of the sequences belonged in group orthogroup 127196 (Table S10), but based on earlier phylogenetic analyses and classification (22) this group includes laccases sensu stricto, laccase-like proteins, L-ascorbate oxidases, melanin synthesis-related proteins, and Fet3 proteins (Fig. S3q). Therefore, the earlier classification and phylogenetic support was used for further separation of AA1 proteins into phylogroups (22).

**AA2.** All but two sequences (Sisni1_487259, Sissu1_1004683) belong in the group OG5_139844 (Table S10). The two *Sistotremastrum* sequences were assigned in the group OG5_233041. However, on the phylogeny these two sequences belong in a small clade with Stehi_182932 and they represent generic peroxidases (Fig. S3r). Therefore, further separation of AA2 genes was not possible and they were all kept as one phylogroup.

**DyP.** OrthoMCL placed most proteins into one group except for a set of eight proteins that were placed in a separate group (Table S10). Because of the small size of this group, DyP were treated as one phylogroup.

**HTP.** OrthoMCL offered little information for separation of HTP proteins in phylogroups (Table S10) and therefore, we used only phylogenetic support to assign phylogroups (Fig. S3t). Proteins Jaaar1_180063 and Exigl1_209010 have an isolated placement and therefore were not included in any phylogroup.

**CRO-AA5.** OrthoMCL was not followed because earlier classification (25) was used to separate the proteins into CRO1, CRO2, CRO6, GLX, GRO3-5 (CRO-WSC) (Fig. S3u).

**GMC.** The GMC proteins represent the most numerous gene family in our analyses. Using phylogenetic information, characterized proteins (Fig. S3v), and OrthoMCL (Table S10) clades representing putative aryl-alcohol oxidases/pyranose dehydrogenases (AAO/PDH, AA3-2), methanol oxidases (AA3-3), and glucose oxidases (GO, AA3-2) were identified. The other clades were not taken into consideration in the PC analyses.

**AA9.** OrthoMCL recognized in total 10 groups and a number of unassigned proteins (no group) (Table S10). Using the phylogenetic analysis (Fig. S3d) the AA9 proteins were separated into 3 phylogroups (A, B, C). Phylogroup A (BS: 82) includes OrthMCL groups 4, 6, 8, and part of group 5. Proteins from group 5 were placed in different clades on the phylogeny and therefore we chose to split them. Phylogroup B (BS: 100) includes proteins from groups 2 and 5. Phylogroup C includes groups 1, 3, and 9 and most of unassigned proteins. Groups 7 and 10 contained single proteins (Calcon 1.55 and Tetni 2.50, respectively) and where placed within the clades containing proteins from groups 1 and 3 and therefore were includes in phylogroup C.

**CE1.** OrthoMCL recognized three groups (Table S10). OG5_193120 included only three proteins, which were present within OG5_141454 (Fig. S3f) and therefore only two phylogroups were recognized.

**CE16.** OrthoMCL recognized in total five groups and two sets of remaining sequences, which were not assigned into groups, but sequences from each set had the same best blast hit (Table S10). These two groups of sequences were placed on two separate well-supported clades on the generated CE16 phylogeny (Fig. S3j). After combining OrthoMCL and phylogenetic information, we recognized three phylogroups (A-C) with good phylogenetic support (BS: 98, 100, 85). Phylogroup A contains OrthoMCL groups OG5_155670, OG5_175897 and the one of the unassigned to a group sequences because sequences from these groups formed paraphyletic clades. Phylogroup B contains only OrthoMCL group OG5_163416 and phylogroup C contains the second set of unassigned sequences and group OG5_176091. The latter group was not separated as a unique group because it contains only three sequences.

**GH11.** Two proteins (GYC_6.28, Gymlu_66885) were the only sequences that were placed in a separate group in the OrthoMCL results (Table S10). However, they were closely related with other GYC, Gymlu proteins and therefore GH11 proteins were used as one phylogroup (Fig. S3k).

**GH28.** OrthoMCL recognized 10 groups and two sets of proteins that were not assigned in any group, but proteins in each of these sets have the same blast hit (Table S10). Sequences from these two unassigned groups formed two distinct phylogenetic groups. Group OG5_209472 contains only one sequence, which was placed with strong support within unassigned sequences (Fig. S3m). Group OG5_169438 contains two sequences that are strongly related to OG5155978. After examination of the tree we separated GH28 proteins into these phylogroups: GH28-A (OG5_155978, OG5_169438, first set of unassigned sequences), GH28-B (OG5_163674, OG5_149950), GH28-C (OG5_188098), GH28-D (OG5_144611, second set of unassigned sequences), GH28-E (OG5_155771), and GH28-F (OG5_130068, OG5_210768).

**GH35.** OrthoMCL information was not used since both groups that were recognized were paraphyletic on the phylogenetic tree.

**GH43.** OrthoMCL detected in total 13 groups and a set of proteins with no assignment, which share the same blast hit result and formed a well-supported clade (Table S10). Furthermore, another set of sequences was not included at all in the OrthoMCL results. blastp searches for those sequences revealed that their best hits are in bacteria. Using the OrthoMCL results and phylogenetic support, we recognized in total 12 groups: GH43-A (OG5_155731), GH43-B (unassigned sequences), GH43-C (OG5_164779), GH43-D (OG5_175874), GH43-E (OG5_176745), GH43-F (OG5_139421, OG5_169300), GH43-G (OG5_149818), GH43-H (unassigned sequences), GH43-I (OG5_149798), GH43-J (OG5_188043), GH43-K (OG5_175862), GH43-L (OG5_187929, OG5_137411).

**GH78.** Two proteins (Stehi1_152826, Stehi1_167237) were placed in a separate category by OrthoMCL, but the family was treated as one phylogroup.

**GH7, GH27, GH62.** Each of these families was represented by one group in OrthoMCL.

**Conditions for the modification of cellulose**

High quality filter paper (Whatman, Grade 2589a, diameter: 140 mm, thickness: 0,43 mm, >97% crystalline cellulose) was partly submerged in 18 ml of modified Highley medium (HG) in 150mm Petri dishes. The liquid medium contained no additional carbon source. a Nylon 66 (polyamide), hydrophilic membrane with pore diameter 1 μm was placed on top of the paper and the fungal inoculum (7 mm) was placed on top of the nylon membrane. The membrane was used to facilitate the removal of the aerial mycelium from the filter paper. The cultures were incubated at 20 or 25^o^C for 40 days in darkness. On day 40, the nylon membrane and the liquid medium were removed and the filter papers were dried at 50^o^C overnight. Control experiments were performed by incubating the filter papers without fungal inoculum.

In order to generate amorphous cellulose, autoclaved strips of the filter paper (approximately 100 mg) were incubated in 50 ml of 3M NaOH solution for 4 hours at room temperature (31). The effect of the commercial mixture of cellulolytic enzymes (Cellic CTec2, Novozyme) on the crystallinity of cellulose was examined using two different enzyme concentrations (C1 and C2) by adding 9 μl and 18 μl of the enzymatic suspension to a final volume of 10 ml 0,1M sodium acetate buffer (pH 4,8), respectively. Strips of autoclaved filter paper (approximately 100 mg) were incubated at 50^o^C (280 rpm) for 6, 12, 24, 48 hours (32). The paper strips from both experiments were rinsed twice with 10 ml Milli-Q water and dried for 24 hours at room temperature. The solution from the enzymatic paper treatments was collected to measure the generated glucose.

**Raman data analyses**

The Raman spectra were analyzed using multivariate curve resolution–alternating least squares (MCR-ALS) (33). The protocol has been developed for FTIR and Raman spectra analysis of biological samples. It uses the whole spectra information to identify components that describe the spectral variation in a dataset. The original files used in the analyses included the spectral region from 1200 to 1550 cm^-1^. This region contains part of the spectral signature of cellulose representing methylene twisting modes and methylene bending vibrations. Changes in this region have been used for the determination of cellulose I, II and amorphous cellulose (34,35). The spectral area between 1250-1491 cm^-1^ was analyzed because the 1200-1250 cm^-1^ region does not contain relevant information and because in the region 1491-1550 cm^-1^ the spectra related to *Gloeophyllum* exhibited a peak likely related to secreted compounds, which would impact the MCR-ALS analysis. Asymmetric least squares (AsLS) baseline correction (λ = 1550 and p = 0,001) was performed on the spectra and the corrected spectra were area-normalized and smoothed using the Savitsky-Golay formalism (order = 1, frame = 3). The preprocessed spectra are shown in Fig. S6. An example of the preprocessed spectra for *Gloeophyllum* and *Phanerochaete* without excluding the 1491-1550 cm^-1^ region is shown in Fig. S7.

**Glucose measurements**

The remaining glucose in the culture filtrates from the FH experiments for the transcriptome and the released glucose during the incubation of the filter paper with Cellic CTec2 was measured using the D-Glucose HK Assay Kit (Megazyme). In order to inactivate all enzymes prior to glucose determination, the samples were heated at 90^o^C for 10 minutes according to the suggestion of the manufacturer. Furthermore, due to the strong color of FH the samples were diluted prior to glucose measurements. The Cellic CTec2 samples were filtrated using 0,2 μm sterile filters prior to glucose determination to remove particles of non-hydrolyzed cellulose.

**Supplementary Figures**

**Fig. S1. Busco results for the seven newly generated genomes.** In total 1335 genes were examined (n: 1335). The majority of genes were found as complete single copies (min. 1167 for *G. confluens*, max. 1303 for *A. pediades*). Duplicated (D), fragmented (F), and missing genes (M) ranged from 10 to 86, 9 to 33 and 5 to 49, respectively.

**Fig. S2. Average number of predicted proteins involved in the degradation of hemicellulose (HE), cellulose (CE) and lignin/xenobiotics (L/X) for the five nutritional strategies.** The y axis shows number of genes and the x axis shows the five nutritional strategies. LD: litter decomposers, BR: brown-rot fungi, WR: white-rot fungi, UWD: uncertain wood decay types, MYC: mycoparasite.

**Fig. S3. RAxML phylogenetic analyses for the 23 gene families used in OrthoMCL, Phylo-PCA and P/A PCA analyses.** a-e: cellulose-decomposition related families, f-p: hemicellulose- and pectin-decomposition related families, q-v: lignin- and xenobiotics- decomposition related families. For subfigures d, j, m, o, q, r, t-v the phylogenetic trees are larger than a page and for those cases a smaller tree with collapsed branches has been used as a guide. On the small trees, collapsed branches colored in blue indicate the part of the tree depicted on the current page. Red circles indicate phylogroups (or whole families) that contributed the most in the Phylo-PCA analyses for cellulose, hemicellulose and lignin/xenobiotics. On the nodes of the trees both separation in phylogroups and OrthoMCL assignments are shown (e.g. GH6-A/137267). For subfigures b and c the presence or absence of genes for *J. argillacea, B. botryosum, C. angulatus, C. cinerea* and brown-rot fungi is indicated. For subfigure q (AA1-MCO) the multiple clades containing Agaricales sequences is shown with red stars. For subfigure r (AA2-Class II peroxidases) the subclassification of sequences in MnP (manganese peroxidases), LiP (lignin peroxidases), VP (versatile peroxidases), a-MnP, (atypical MnP), a-VP (atypical-VP), G (generic peroxidase), U (uncertain type) is shown. Red stars indicate the multiple origins of high ligninolytic potential (HLP) peroxidases (LiP, VP, a-VP) across the phylogeny. Notice that such events have taken place four times in Polyporales and once in Agaricales (*G. marginata, A. pediades*). For subfigure v names of sequences in red font represent characterized GenBank sequences (not derived from the genomic data used in the study).

**Fig. S4.** **Variables coordinates from the phylogroups principal component analyses for the cellulose (a), hemicellulose (b), and lignin/xenobiotics (c) datasets.** Stars in (b) show variables (phylogroups) that received PC values ≥ 0,20 and ≤ -0,20 and belong to families assigned to pectin (yellow), xylan (black), cutin (orange), and blue (xyloglucan). LD: litter decomposers, BR: brown-rot fungi, WR: white-rot fungi, UWD: uncertain wood decay types, MYC: mycoparasite. Gene families acronyms explained in Table S6.

**Fig. S5. P/A PCA variables coordinates**. P-A PCA plot showing the coordinates of the variables (phylogroups) for the 68 phylogroups presence /absence dataset showing the clustering of species based on nutritional strategy. LD: litter decomposers, BR: brown-rot fungi, WR: white-rot fungi, UWD: uncertain wood decay types, MYC: mycoparasite. Acronyms of gene families are explained in Table S6.

**Fig. S6. Preprocessed Raman spectra of cellulose.** The spectra of cellulose are shown for the control, fungi-colonized, NaOH treatment, and enzymatic treatments samples after baseline correction, area normalization and use of S-G filter (Savitzky-Golay smoothing).


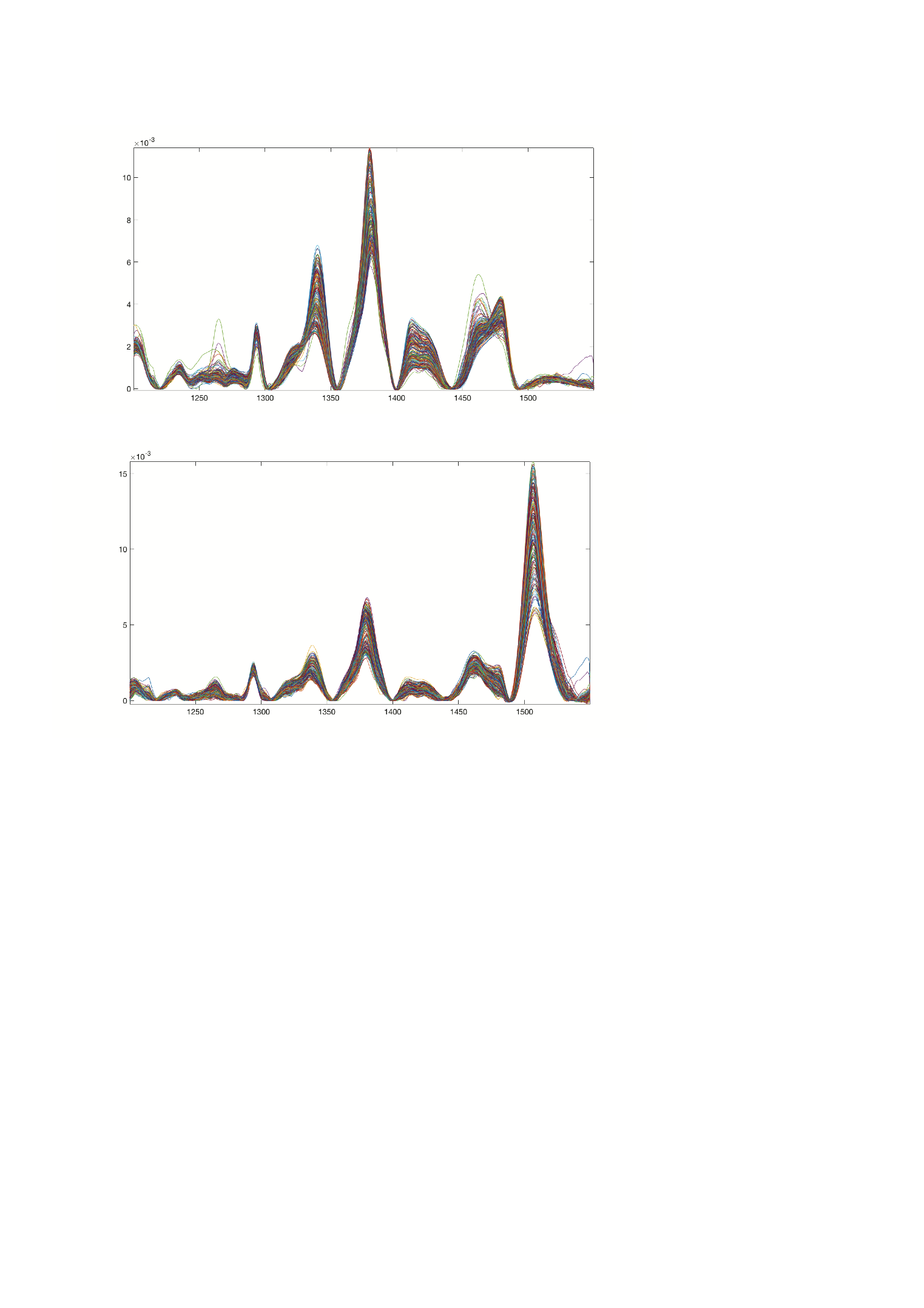


**Fig. S7. Cellulose spectra (1250-1550 cm^-1^**) **for samples colonized by the *Phanerochaete* and *Gloeophyllum* strains*.*** The *Phanerochaete* (upper) and the *Gloeophyllum* (lower) related spectra are shown after baseline correction, area normalization and use of S-G filter. The spectral area 1491-1550 cm-1 has been included. Notice the prominent peak seen only for *Gloeophyllum*-related spectra at around 1510 cm^-1^.

**Fig. S8.** **Glucose generated from cellulose during Cellic CTec2 treatment.** Concentration of glucose (g/l, y axis) generated during enzymatic treatment of cellulose with 9μl (C1) and 18μl (C2) of enzymatic solution per 10 ml of incubation solution (C1 and C2, respectively) and for four time points (x axis, hours). The measurements are the result of three replicates. The bars show standard error (*n*=3). The enzymatic solution contained a small, measurable amount of glucose, which was subtracted from the total amount of glucose measured.

**Supplementary Tables**

**Table S1**. **Newly sequenced fungal strains.** M: monokaryon, D: dikaryon, T: temperature preference. Acronyms in parentheses indicate the name appearing on the proteins sequence IDs.

| Species | Acronym used | Strain | Sexual stage | T (^o^C) | Origin | Habitat |
| --- | --- | --- | --- | --- | --- | --- |
| ***Agrocybe pediades*** | AGP (Agpe) | CBS 102.39 | M | 20 | unknown | Grasslands (occasionally on wood chips or dung) |
| ***Psilocybe cf. subviscida*** | PSS (Psisu) | CBS 101986 | M | 20 | Noord-Holland Amsterdam  May 1997 | Grasslands, dung, humus |
| ***Tetrapyrgos nigripes*** | TEN (Tetni) | CBS 291.85 | D | 25 | Pinery Provincial Park, Ontario Canada,  August 1983 | Leaf litter and twigs in hardwood forests |
| ***Gymnopus confluens*** | GYC (Gymco) | CBS 406.79 | D | 20 | France  October 1963 | Conifer and hardwood litter |
| ***Tricholomella constricta*** | TRC (Calcon) | CBS 661.87 | D | 20 | France | Pastures, hardwood litter |
| ***Leucoagaricus leucothites*** | LEL (Lepsp) | CBS 146.42 | D | 25 | Sweden | Pastures, occasionally in woodlands |
| ***Coprinellus angulatus*** | COA (Copan) | CBS 175.51 | M | 20 | Versailles  France,  7 Aug. 1950 | Regenerating ecosystems after forest fires |

**Table S2. Genomic characteristics of the newly sequenced genomes.**

| **Species acronym** | **GYC** | **TEN** | **TRC** | **COA** | **LEL** | **AGP** | **PSS** |
| --- | --- | --- | --- | --- | --- | --- | --- |
| **Total sequence length (Mb)** | 60,1 | 99,1 | 44,4 | 59,3 | 47,3 | 45,1 | 53,0 |
| **# contigs** | 855 | 702 | 158 | 273 | 144 | 70 | 68 |
| **Largest contig (Mb)** | 1,09 | 1,50 | 2,75 | 3,71 | 3,76 | 3,93 | 7,64 |
| **N50 (Mb)** | 0,18 | 0,34 | 0,87 | 0,75 | 2,10 | 2,72 | 2,90 |
| **mRNA reads mapped % ^*^** | 92,0 | 92,9 | 94,3 | 92,6 | 92,9 | 93,5 | 91,9 |
| **L50** | 83 | 80 | 16 | 21 | 9 | 7 | 6 |
| **Number of genes** | 15320 | 18803 | 10603 | 15072 | 11537 | 12919 | 13686 |
| **Mean gene length** | 2305 | 2197 | 2437 | 2235 | 2469 | 2359 | 23941 |
| **Mean exon length** | 255 | 264 | 273 | 309 | 269 | 280 | 289 |
| **Mean intron length** | 80 | 84 | 76 | 77 | 74 | 73 | 80 |
| **Mean exons per mRNA** | 7 | 7 | 7 | 6 | 7 | 7 | 7 |
| **Mean introns per mRNA** | 6 | 6 | 6 | 5 | 6 | 6 | 6 |

^*^ For the dikaryotic strains the mRNA reads were mapped both on primary and alternative contigs^.^

**Table S3**. **Published genomes used in the study.** The version of the genome used is shown next to the species name.

| **Species** | **Acronym** | **Order** | **Publication** |
| --- | --- | --- | --- |
| ***Agaricus bisporus var. bisporus* (v.2)** | Agabi | Agaricales | Morin et al., 2012 |
| ***Coprinopsis cinerea* (v.1)** | Copci | Agaricales | Stajich et al., 2010 |
| ***Cylindrobasidium torrendii* (v.1)** | Cylto | Agaricales | Floudas et al., 2015 |
| ***Galerina marginata* (v.1)** | Galma | Agaricales | Riley et al., 2014 |
| ***Gymnopus luxurians* (v.1)** | Gymlu | Agaricales | Kohler et al., 2015 |
| ***Schizophyllum commune* (v.3)** | Schco | Agaricales | Ohm et al., 2010 |
| ***Plicaturopsis crispa* (v.1)** | Plicr | Amylocorticiales | Kohler et al., 2015 |
| ***Auricularia subglabra* (v.3_1)** | Aurde | Auriculariales | Floudas et al., 2012 |
| ***Exidia glandulosa* (v.1)** | Exigl | Auriculariales | Nagy et al., 2015 |
| ***Coniophora puteana* (v.1)** | Conpu | Boletales | Floudas et al., 2012 |
| ***Hydnomerulius pinastri* (v.2)** | Hydpi | Boletales | Kohler et al., 2015 |
| ***Serpula lacrymans* (S7-3, v.2)** | Serla | Boletales | Eastwood et al., 2011 |
| ***Botryobasidium botryosum* (v.1)** | Botbo | Cantharellales | Riley et al., 2014 |
| ***Punctularia strigoso-zonata* (v.1)** | Punst | Corticiales | Floudas et al., 2012 |
| ***Calocera cornea* (v.1)** | Calco | Dacrymycetales | Nagy et al., 2015 |
| ***Calocera viscosa* (v.1)** | Calvi | Dacrymycetales | Nagy et al., 2015 |
| ***Dacryopinax primogenitus* (v.1)** | Dacsp | Dacrymycetales | Floudas et al., 2012 |
| ***Gloeophyllum trabeum* (v.1)** | Glotr | Gloeophyllales | Floudas et al., 2012 |
| ***Neolentinus lepideus* (v.1)** | Neole | Gloeophyllales | Nagy et al., 2015 |
| ***Fomitiporia mediterranea* (v.1)** | Fomme | Hymenochaetales | Floudas et al., 2012 |
| ***Jaapia argillacea* (v.1)** | Jaaar | Jaapiales | Riley et al., 2014 |
| ***Bjerkandera adusta* (v.1)** | Bjead | Polyporales | Binder et al., 2013 |
| ***Daedalea quercina* (v.1)** | Daequ | Polyporales | Nagy et al., 2015 |
| ***Dichomitus squalens* (v.1)** | Dicsq | Polyporales | Floudas et al., 2012 |
| ***Fomitopsis pinicola* (v.3)** | Fompi | Polyporales | Floudas et al., 2012 |
| ***Phlebia brevispora* (v.1)** | Phlbr | Polyporales | Binder et al., 2013 |
| ***Ganoderma sp.* (v.1)** | Gansp | Polyporales | Binder et al., 2013 |
| ***Gelatoporia subvermispora* (v.1)** | Cersu | Polyporales | Fernandez-Fueyo et al., 2012 |
| ***Trametes versicolor* (v.1)** | Trave | Polyporales | Floudas et al., 2012 |
| ***Wolfiporia coccos* (v.1)** | Wolco | Polyporales | Floudas et al., 2012 |
| ***Peniophora sp.* (v.1)** | Peni | Russulales | Nagy et al., 2015 |
| ***Stereum hirsutum* (v.1)** | Stehi | Russulales | Floudas et al., 2012 |
| ***Sistotremastrum niveocremeum* (v.1)** | Sisni | Trechisporales | Nagy et al., 2015 |
| ***Sistotremastrum suecicum* (v.1)** | Sissu | Trechisporales | Nagy et al., 2015 |
| ***Tremella mesenterica* (v.1)** | Treme | Tremellales | Floudas et al., 2012 |

**Table S4.** **Pfam, InterPro and SSF identifiers used for data collection.** (P) indicates that further separation of the dataset relied on a phylogenetic tree and previously annotated data. * identifiers that recovered a larger set of proteins. ** gene families for which blast searches were used in addition to identifiers. CAZy classification has been followed. CE, HE, L/X: cellulose-, hemicellulose-, and lignin/xenobiotics-decomposition genes families, respectively.

| Functional Category | Gene family | IPR, Pfam, SSF Identifiers |
| --- | --- | --- |
| CE | AA8-AA12 | SSF49344 ** |
| CE | AA8 | SSF49344 ** |
| CE | AA9 | PF03443 |
| CE | ALE | PF01263 |
| CE | GH1 | PF00232 |
| CE | GH12 | PF01670 |
| CE | GH43 | PF04616 |
| CE | GH6 | PF01341 |
| CE | GH7 | PF00840 |
| CE | GH9 | PF00759 |
| CE | GH5_5 | PF00150 * |
| CE, HE | GH3 | PF00933 / PF01915 (P) |
| HE | CE1 | IPR010126 / SSF53474 ** |
| HE | CE12 | IPR013830 * |
| HE | CE16 | IPR013830 * |
| HE | CE5 | PF01083 |
| HE | CE8 | PF01095 |
| HE | GH10 | PF00331 |
| HE | GH11 | PF00457 |
| HE | GH115 | PF15979 ** |
| HE | GH2 | IPR006102 / IPR008979 * |
| HE | GH27 | IPR000111 * |
| HE | GH28 | PF00295 |
| HE | GH29 | PF01120 |
| HE | GH35 | PF01301 |
| HE | GH5_7 | PF00150 * |
| HE | GH51 | PF06964 |
| HE | GH53 | PF07745 |
| HE | GH62 | PF03664 |
| HE | GH74 | SSF110296 * |
| HE | GH78 | IPR008928 * |
| HE | GH88 | IPR008928 * |
| HE | GH95 | IPR008928 * |
| L/X | AA1 MCO | PF07732 / PF07731 / PF00394 (P) |
| L/X | AA2 POD | PF00141 (P) |
| L/X | AA3_1, AA3_2, AA3_3 GMC | PF05199 / PF00732 (P) |
| L/X | AA3_4, pyranose oxidase | IPR012814 |
| L/X | AA5 CRO | PF07250 (P) |
| L/X | DyP | IPR006314 |
| L/X | HTP | IPR000028 |

**Table S5. Gene copy numbers in 44 gene families across 42 genomes.** Gene families are separated in functional categories related to cellulose, hemicellulose (including pectin), and lignin/xenobiotics decomposition. Nutritional strategies: MYC, mycoparasite; WR, white rot; BR, brown rot; LD, litter decomposer; UWD, uncertain wood decay type. Cells with zero genes are shown in blue. For each functional category the total number of genes per species is shown in the right end of the category and the total number of all genes per species are shown in the right end of the table. The color gradient (yellow to red) highlights the gene richness for each category or for the total number of genes per species. The table is provided as a separate file in Dryad (doi.org/10.5061/dryad.pk0p2ngk1).

**Table S6. Enzymatic activities associated with gene families and PCA values for A-PCA, Phylo-PCA, and P/A-PCA for the first two components.** For A-PCA and Phylo-PCA values ≥ 0,2 and ≤ 0,20 and for P/A-PCA values ≥ 0,15 and ≤ 0,15 are shown in bold and red. Phylogroups that received high values for both Phylo-PCA and P/A-PCA are highlighted in green; phylogroups that received high values only in Phylo-PCA or P/A-PCA are highlighted in blue or grey, respectively. Potential enzymatic activities for each family are shown on the first column. CE and L/X indicate gene families related to cellulose and lignin/xenobiotics decomposition. Hemicellulose (HE) decomposition gene families are further separated into groups related to pectin- (PE), xylan- (XYL), xyloglucan- (XG), mannan- (MAN), and cutin- (CUT) decomposition.

| **Enzymatic activities** | **Gene family** | **A-PCA** | | **Phylogroup** | **Phylo-PCA** | | **P/A-PCA** | |
| --- | --- | --- | --- | --- | --- | --- | --- | --- |
|  |  | PC1 | PC2 |  | PC1 | PC2 | PC1 | PC2 |
| lytic polysaccharide monooxygenase (CE) | **AA9** | **0,702** | 0,172 | **AA9_A** | **0,488** | -0,036 | **-0,18** | 0,032 |
|  |  |  |  | **AA9_B** | **0,434** | 0,119 | **-0,167** | 0,024 |
|  |  |  |  | **AA9_C** | **0,434** | 0,032 | -0,107 | 0,084 |
| cellobiohydrolase (CE) | **GH7** | **0,526** | 0,09 | **GH7** | **0,459** | -0,073 | **-0,202** | -0,017 |
| cellobiohydrolase (CE) | **GH6** | **0,322** | 0,13 | **GH6_A** | **0,253** | -0,191 | **-0,174** | 0,025 |
|  |  |  |  | GH6_B | 0,021 | 0,069 | -0,016 | -0,013 |
| endoglucanase (CE) | **GH5-5** | 0,186 | **-0,446** | **GH5_5_A** | **0,226** | **0,238** | **-0,25** | -0,104 |
|  |  |  |  | **GH5_5_B** | -0,045 | **-0,708** | -0,004 | **0,224** |
|  |  |  |  | **GH5_5_C** | 0,113 | -0,088 | **-0,172** | -0,076 |
| endoglucanase (CE) | **GH12** | 0,13 | **-0,705** | **GH12_A** | 0,034 | **-0,474** | -0,031 | **0,159** |
|  |  |  |  | **GH12_B** | 0,087 | -0,166 | -0,136 | **0,226** |
| cellobiose dehydrogenase (CE) | **CDH** | **0,218** | -0,009 | **CDH** | 0,178 | -0,104 | **-0,18** | 0,032 |
| iron reducing protein (CE) | **AA8** | 0,114 | **-0,205** | **AA8_A** | 0,001 | **-0,218** | 0,009 | **0,259** |
|  |  |  |  | AA8_B | 0,032 | -0,131 | 0,042 | -0,003 |
|  |  |  |  | AA8_C | 0,015 | 0,071 | -0,016 | -0,013 |
| pyrroloquinoline quinone-dependent oxidoreductase (CE) | **AA8-AA12** | 0,039 | **0,362** | AA8-AA12 | 0,05 | 0,197 | -0,001 | -0,112 |
| β-glucosidase (CE) | **GH1** | 0,091 | -0,125 | **-** | **-** | **-** | **-** | **-** |
| β-glucosidase (CE) | **GH3** | 0,05 | -0,199 | **-** | **-** | **-** | **-** | **-** |
| aldose epimerase (CE) | **ALE** | 0,043 | -0,122 | **-** | **-** | **-** | **-** | **-** |
| endoglucanase (CE) | **GH9** | 0,026 | 0,076 | **-** | **-** | **-** | **-** | **-** |
| various activities (PE) | **GH43** | **0,371** | -0,073 | **GH43-A** | 0,066 | 0,179 | -0,043 | **0,196** |
|  |  |  |  | GH43-B | 0,077 | -0,063 | -0,111 | -0,119 |
|  |  |  |  | GH43-C | 0,011 | -0,121 | -0,035 | -0,148 |
|  |  |  |  | GH43-D | 0,036 | 0,007 | -0,021 | 0,001 |
|  |  |  |  | GH43-E | 0,019 | -0,065 | -0,015 | -0,018 |
|  |  |  |  | GH43-F | 0,054 | -0,016 | -0,001 | -0,041 |
|  |  |  |  | **GH43-G** | **0,255** | 0,026 | -0,145 | 0,006 |
|  |  |  |  | GH43-H | 0,116 | 0,075 | -0,098 | 0,133 |
|  |  |  |  | GH43-I | -0,089 | -0,007 | 0,106 | -0,053 |
|  |  |  |  | **GH43-J** | 0,161 | -0,086 | **-0,184** | -0,113 |
|  |  |  |  | GH43-K | 0,107 | -0,049 | -0,097 | -0,012 |
|  |  |  |  | **GH43-L** | **0,350** | 0,039 | -0,14 | -0,03 |
| pectinase (PE) | **GH28** | **0,242** | **-0,28** | GH28-A | 0,163 | 0,111 | -0,055 | 0,091 |
|  |  |  |  | **GH28-B** | 0,087 | **0,226** | -0,064 | **0,208** |
|  |  |  |  | GH28-C | 0,093 | -0,007 | -0,116 | 0,004 |
|  |  |  |  | **GH28-D** | 0,096 | **0,252** | -0,022 | **0,309** |
|  |  |  |  | GH28-E | 0,097 | -0,029 | -0,061 | 0,006 |
|  |  |  |  | **GH28-F** | 0,164 | 0,18 | -0,025 | **0,21** |
| pectin methylesterase (PE) | **CE8** | **0,243** | **-0,206** | **CE8-A** | **0,288** | -0,039 | -0,135 | 0,026 |
|  |  |  |  | **CE8-B** | 0,055 | 0,175 | -0,041 | **0,224** |
| pectin acetylesterase (PE) | **CE12** | **0,337** | 0,092 | **CE12-A** | **0,263** | -0,096 | **-0,24** | -0,112 |
|  |  |  |  | **CE12-B** | 0,168 | -0,087 | **-0,176** | -0,119 |
| β-galactosidase (PE) | **GH35** | **0,279** | -0,131 | **GH35** | **0,270** | 0,095 | -0,031 | 0,039 |
| α-L-rhamnosidase (PE) | **GH78** | **0,223** | **-0,32** | **GH78** | **0,242** | **0,283** | -0,131 | **0,249** |
| acetylesterase (XYL) | **CE16** | **0,247** | -0,195 | CE16-A | 0,159 | 0,172 | -0,031 | 0,039 |
|  |  |  |  | **CE16-B** | **0,227** | 0,108 | -0,02 | 0,049 |
|  |  |  |  | **CE16-C** | 0,196 | 0,179 | -0,141 | **0,229** |
| acetyl-xylan esterase (XYL) | **CE1** | **0,239** | **0,287** | **CE1-A** | 0,172 | **-0,251** | **-0,181** | -0,06 |
|  |  |  |  | CE1-B | 0,152 | -0,011 | -0,108 | 0,008 |
| xylanase (XYL) | **GH11** | **0,241** | **0,473** | **GH11** | **0,201** | **-0,449** | **-0,15** | **-0,184** |
| α-L-arabinofuranosidase (XYL) | **GH62** | 0,148 | **0,243** | **GH62** | 0,129 | **-0,254** | **-0,159** | **-0,2** |
| α-galactosidase (MAN) | **GH27** | **0,234** | **-0,214** | **GH27** | **0,223** | 0,14 | -0,032 | 0,095 |
| cutinase (CUT) | **CE5** | **0,333** | **0,443** | **CE5-A** | **0,202** | **-0,442** | **-0,152** | **-0,211** |
|  |  |  |  | **CE5-B** | 0,143 | -0,108 | **-0,155** | -0,017 |
| α-L-arabinofuranosidase (XG) | **GH51** | 0,178 | -0,084 | - | - | - | - | - |
| xylanase (XYL) | **GH10** | 0,159 | 0,117 | - | - | - | - | - |
| α-1,2-L-fucosidase (XG) | **GH95** | 0,139 | -0,068 | - | - | - | - | - |
| α-L-fucosidase (XG) | **GH29** | 0,115 | -0,13 | - | - | - | - | - |
| β-xylosidase (XYL) | **GH3** | 0,108 | -0,121 | - | - | - | - | - |
| xyloglucanase (XG) | **GH74** | 0,106 | 0,05 | - | - | - | - | - |
| β-mannosidase (MAN) | **GH2** | 0,084 | -0,129 | - | - | - | - | - |
| endo-β-1,4-galactanase (PE) | **GH53** | 0,083 | -0,072 | - | - | - | - | - |
| β-glucuronyl hydrolase (PE) | **GH88** | 0,081 | -0,085 | - | - | - | - | - |
| mannanase (MAN) | **GH5-7** | 0,08 | 0,089 | - | - | - | - | - |
| xylan α-1,2-glucuronidase (XYL) | **GH115** | 0,023 | -0,069 | - | - | - | - | - |
| Class II peroxidase (L/X) | **AA2** | **0,715** | **0,630** | **AA2** | **0,464** | **0,495** | **-0,217** | 0,083 |
| laccase (L/X) | **AA1** | **0,254** | -0,083 | **AA1-A** | **0,367** | -0,03 | -0,116 | 0,122 |
|  |  |  |  | AA1-B | -0,097 | 0,199 | 0,067 | **0,188** |
| dye decolorizing peroxidase (L/X) | **DyP** | **0,425** | **-0,331** | **DyP** | **0,303** | 0,034 | **-0,2** | -0,063 |
| heme thiolate peroxidase (L/X) | **HTP** | **0,278** | **-0,625** | HTP-A | 0,132 | 0,098 | -0,107 | 0,084 |
|  |  |  |  | HTP-B | 0,01 | 0,04 | -0,02 | 0,049 |
|  |  |  |  | HTP-C | 0,163 | 0,167 | -0,091 | 0,015 |
|  |  |  |  | **HTP-D** | 0,018 | **0,266** | -0,012 | -0,021 |
|  |  |  |  | **HTP-E** | -0,076 | **0,21** | 0,055 | -0,007 |
|  |  |  |  | **HTP-F** | **0,21** | **-0,673** | -0,113 | **-0,188** |
| hydrogen peroxide production (L/X) | **AA5** | **0,273** | -0,109 | **AA5-CRO1** | -0,027 | **-0,206** | 0,031 | -0,133 |
|  |  |  |  | AA5-CRO2 | 0,154 | -0,034 | -0,139 | 0,046 |
|  |  |  |  | AA5-CRO6 | 0,105 | 0,07 | -0,031 | 0,039 |
|  |  |  |  | **AA5-GLX** | **0,305** | -0,03 | **-0,238** | 0,053 |
|  |  |  |  | AA5 CRO-WSC | 0,14 | -0,03 | **-0,182** | 0,051 |
| hydrogen peroxide production (L/X) | **GMC** | **0,284** | **-0,289** | **AAO-PDH** | **0,526** | **-0,208** | **-0,153** | 0,009 |
|  |  |  |  | MOX | 0,107 | 0,035 | -0,031 | 0,039 |
|  |  |  |  | GO | 0,135 | 0,107 | 0,000 | 0,000 |
| hydrogen peroxide production (L/X) | **PO** | 0,103 | -0,028 | - | - | - | - | - |

**Table S7. PERMANOVA analyses results.** The upper table shows the statistical support for the differences between the four major nutritional strategies (LD: litter decomposers, WR: white-rot fungi, BR: brown-rot fungi, UWD: fungi with uncertain wood decay type) and between the two most densely sampled orders (Aga: Agaricales, Pol: Polyporales) for the four datasets (CE: cellulose-decomposition phylogroups, HE: hemicellulose-decomposition phylogroups, L/X: lignin/xenobiotics-decomposition phylogroups, P/A: presence/absence dataset). A separate comparison between LDs and WR fungi for the ligninolytic class II peroxidases is shown at the right of the upper table. The lower table shows the statistical support for the separation of nutritional strategies and for the separation of the two most densely sampled orders along PC1 and PC2 for the PC analyses of all four datasets (CE: cellulose-decomposition phylogroups, HE: hemicellulose-decomposition phylogroups, L/X: lignin/xenobiotics-decomposition phylogroups, P/A: presence/absence dataset). For all pairwise comparisons Bonferroni corrected p-values are shown. The table is provided as a separate file in Dryad (doi.org/10.5061/dryad.pk0p2ngk1).

**Table S8**. **Total number of replicates and number of Raman collected spectra for the controls, fungi-colonized samples, enzyme-treated samples and** **NaOH-treated samples.**

| **Sample** | **Acronym** | **No. of spectra per replicate** | **No. of replicates** | **Total no. of spectra** |
| --- | --- | --- | --- | --- |
| **original paper (non-autoclaved)** | OP | 66 | 3 | 198 |
| **autoclaved paper (non-incubated)** | AUP | 66 | 3 | 198 |
| **control at 20 C** | C20 | 66 | 3 | 198 |
| **control at 25 C** | C25 | 66 | 3 | 198 |
| ***Gloeophyllum* sp.** | GL | 66 | 3 | 198 |
| ***Phanerochaete* sp.** | PL | 66 | 3 | 198 |
| ***Agrocybe pediades*** | AGP | 66 | 3 | 198 |
| ***Coprinellus angulatus*** | COA | 66 | 3 | 198 |
| ***Gymnopus confluens*** | GYC | 66 | 3 | 198 |
| ***Leucoagaricus leucothites*** | LEL | 66 | 3 | 198 |
| ***Psilocybe cf subviscida*** | PSS | 66 | 3 | 198 |
| ***Tetrapyrgos nigripes*** | TEN | 66 | 3 | 198 |
| ***Tricholomella constricta*** | TRC | 66 | 3 | 198 |
| **CellicCTec2**  **C2-24h** | EnzC2_24h | 18 | 7 | 126 |
| **CellicCTec2**  **C2-48h** | EnzC2_48h | 18 | 7 | 126 |
| **NaOH** | NaOH3M4h | 18 | 7 | 126 |

**Table S9. Subclassification of AA1, AA5, GH3, and AA3 across the 42 genomes.** The color of the cells (yellow to orange) is in accordance to the number of genes found in each species (yellow for one copy and orange to the highest number of copies). The table is provided as a separate file in Dryad (doi.org/10.5061/dryad.pk0p2ngk1).

**Table S10. OrthoMCL assignments for 23 gene families.** If a protein was assigned to a phylogroup it is shown to the right side of the table. The table is provided as a separate file in Dryad (doi.org/10.5061/dryad.pk0p2ngk1).

**References**

1. Davidson EA, Galloway LF, Strand MK. Assessing available carbon: Comparison of techniques across selected forest soils. Commun Soil Sci Plant Anal. 1987;18(1):45–64.

2. Chin C-S, Alexander DH, Marks P, Klammer AA, Drake J, Heiner C, et al. Nonhybrid, finished microbial genome assemblies from long-read SMRT sequencing data. Nat Methods. 2013;10(6):563–9.

3. Chin C-S, Peluso P, Sedlazeck FJ, Nattestad M, Concepcion GT, Clum A, et al. Phased diploid genome assembly with single-molecule real-time sequencing. Nat Methods. 2016;13(12):1050–4.

4. Gurevich A, Saveliev V, Vyahhi N, Tesler G. QUAST: Quality assessment tool for genome assemblies. Bioinformatics. 2013;29(8):1072–5.

5. Simão FA, Waterhouse RM, Ioannidis P, Kriventseva E V., Zdobnov EM. BUSCO: Assessing genome assembly and annotation completeness with single-copy orthologs. Bioinformatics. 2015;31(19):3210–2.

6. Kim D, Langmead B, Salzberg SL. HISAT: A fast spliced aligner with low memory requirements. Nat Methods. 2015;12(4):357–60.

7. Li H, Handsaker B, Wysoker A, Fennell T, Ruan J, Homer N, et al. The Sequence Alignment/Map format and SAMtools. Bioinformatics. 2009;25(16):2078–9.

8. Pertea M, Pertea GM, Antonescu CM, Chang TC, Mendell JT, Salzberg SL. StringTie enables improved reconstruction of a transcriptome from RNA-seq reads. Nat Biotechnol. 2015;33(3):290–5.

9. Lagesen K, Hallin P, Rødland EA, Stærfeldt HH, Rognes T, Ussery DW. RNAmmer: Consistent and rapid annotation of ribosomal RNA genes. Nucleic Acids Res. 2007;35(9):3100–8.

10. Tarailo-Graovac M, Chen N. Using RepeatMasker to identify repetitive elements in genomic sequences. Curr Protoc Bioinforma. 2009;Chapter 4(March):Unit 4.10.

11. Smith CD, Edgar RC, Yandell MD, Smith DR, Celniker SE, Myers EW, et al. Improved repeat identification and masking in Dipterans. Gene. 2007;389(1):1–9.

12. Yandell M, Holt C. MAKER2: an annotation pipeline and genome-database management tool for second-generation genome projects. BMC Bioinformatics. 2011;12(1):491.

13. Ter-hovhannisyan V, Lomsadze A, Chernoff YO, Borodovsky M. Gene prediction in novel fungal genomes using an ab initio algorithm with unsupervised training. Genome Res. 2008;18:1979–90.

14. Stanke M, Waack S. Gene prediction with a hidden Markov model and a new intron submodel. Bioinformatics. 2003;19(SUPPL. 2):215–25.

15. Korf I. Gene finding in novel genomes. BMC Bioinformatics. 2004;5:1–9.

16. Hoff KJ, Lange S, Lomsadze A, Borodovsky M, Stanke M. BRAKER1: Unsupervised RNA-Seq-based genome annotation with GeneMark-ET and AUGUSTUS. Bioinformatics. 2016;32(5):767–9.

17. Finn RD, Bateman A, Clements J, Coggill P, Eberhardt RY, Eddy SR, et al. Pfam: The protein families database. Nucleic Acids Res. 2014;42(D1):222–30.

18. El-Gebali S, Mistry J, Bateman A, Eddy SR, Luciani A, Potter SC, et al. The Pfam protein families database in 2019. Nucleic Acids Res. 2019;47(D1):D427–32.

19. Jones P, Binns D, Chang HY, Fraser M, Li W, McAnulla C, et al. InterProScan 5: Genome-scale protein function classification. Bioinformatics. 2014;30(9):1236–40.

20. Kohler A, Kuo A, Nagy LG, Morin E, Barry KW, Buscot F, et al. Convergent losses of decay mechanisms and rapid turnover of symbiosis genes in mycorrhizal mutualists. Nat Genet. 2015;47(4):410–5.

21. Floudas D, Binder M, Riley R, Barry K, Blanchette RA, Henrissat B, et al. The paleozoic origin of enzymatic lignin decomposition reconstructed from 31 fungal genomes. Science (80- ). 2012;336(6089):1715–9.

22. Kues U, Ruhl M. Multiple Multi-Copper Oxidase Gene Families in Basidiomycetes – What for? Curr Genomics. 2011;12(2):72–94.

23. Ruiz-Dueñas FJ, Martínez AT. Structural and Functional Features of Peroxidases with a Potential as Industrial Biocatalysts BT - Biocatalysis Based on Heme Peroxidases: Peroxidases as Potential Industrial Biocatalysts. In: Torres E, Ayala M, editors. Berlin, Heidelberg: Springer Berlin Heidelberg; 2010. p. 37–59.

24. Ferreira P, Carro J, Serrano A, Martinez AT. A survey of genes encoding H2O2-producing GMC oxidoreductases in 10 Polyporales genomes. Mycologia. 2015;107(6):1105–19.

25. Kersten P, Cullen D. Copper radical oxidases and related extracellular oxidoreductases of wood-decay Agaricomycetes. Fungal Genet Biol. 2014;72:124–30.

26. Fischer S, Brunk BP, Chen F, Gao X, Harb OS, Iodice JB, et al. Using OrthoMCL to assign proteins to OrthoMCL-DB groups or to cluster proteomes into new ortholog groups. Curr Protoc Bioinforma. 2011;(SUPPL.35):1–19.

27. Miller MA, Pfeiffer W, Schwartz T. Creating the CIPRES Science Gateway for inference of large phylogenetic trees. 2010 Gatew Comput Environ Work GCE 2010. 2010;

28. Stamatakis A. RAxML version 8: A tool for phylogenetic analysis and post-analysis of large phylogenies. Bioinformatics. 2014;30(9):1312–3.

29. Katoh K, Rozewicki J, Yamada KD. MAFFT online service: multiple sequence alignment, interactive sequence choice and visualization. Brief Bioinform. 2017;(June):1–7.

30. Larsson A. AliView: A fast and lightweight alignment viewer and editor for large datasets. Bioinformatics. 2014;30(22):3276–8.

31. Jiao C, Xiong J. Accessibility and morphology of cellulose fibres treated with sodium hydroxide. BioResources. 2014;9(4):6504–13.

32. Rodrigues AC, Haven MØ, Lindedam J, Felby C, Gama M. Celluclast and Cellic^®^ CTec2: Saccharification/fermentation of wheat straw, solid-liquid partition and potential of enzyme recycling by alkaline washing. Enzyme Microb Technol. 2015;79–80:70–7.

33. Felten J, Hall H, Jaumot J, Tauler R, De Juan A, Gorzsás A. Vibrational spectroscopic image analysis of biological material using multivariate curve resolution-alternating least squares (MCR-ALS). Nat Protoc. 2015;10(2):217–40.

34. Ambjörnsson HA, Schenzel K, Germgård U. Carboxymethyl cellulose produced at different mercerization conditions and characterized by nir ft raman spectroscopy in combination with multivariate analytical methods. BioResources. 2013;8(2):1918–32.

35. Schenzel K, Fischer S, Brendler E. New method for determining the degree of cellulose I crystallinity by means of FT Raman spectroscopy. Cellulose. 2005;12(3):223–31.
